# Foundations of climate change denial: Anti-environmentalism and anti-science

**DOI:** 10.1371/journal.pone.0334544

**Published:** 2025-11-12

**Authors:** Peter J. Jacques, Riley E. Dunlap

**Affiliations:** 1 Rechnitz Family/Urban Coast Institute Endowed Chair in Marine and Environmental Law and Policy, Department of Political Science and Sociology, Monmouth University, New Jersey, United States of America; 2 Dresser Professor and Regents Professor of Sociology Emeritus, Oklahoma State University, Stillwater, Oklahoma, United States of America; The University of Edinburgh, UNITED KINGDOM OF GREAT BRITAIN AND NORTHERN IRELAND

## Abstract

Despite a longstanding scientific consensus about the reality of anthropogenic global warming (AGW), a climate change countermovement (CCCM) has worked to undermine and cast doubt on climate science for over three decades. The CCCM is a coalition led by fossil fuel corporations and their advocacy organizations, far-right conservative think tanks (CTTs), conservative foundations and a few dissenting scientists that has successfully thwarted domestic mitigation policies and international agreements aimed at reducing greenhouse gas emissions (GHGs). Social science investigations into the CCCM have become increasingly sophisticated and have provided key insights into the content and influence of AGW denial narratives. Denial narratives reject the basic findings of climate science: the earth is warming (trend denial), largely due to human actions (attribution denial), producing harmful impacts (impact denial), *and* mitigation policies are ineffective or harmful (policy denial). These narratives cast the integrity of climate science and scientists in doubt; yet a fine-grained analysis of denial narratives has not been conducted. To fill this gap, we analyze the content of 108 books that reject climate science using a two-stage content analysis approach: first, a deductive approach to identify denial claims in the books, and second an inductive approach to analyze the larger semantic ecosystems surrounding the claims. We confirm the major narratives that have been identified in prior research, but discover a consistent, underlying anti-environmentalism along with a rejection of “impact science” that highlights the negative effects of industrial production. These two meta-themes challenge reflexive modernization, which relies on scientific knowledge and global environmentalism to solve environmental problems. This reflects a deep “anti-reflexivity” employed to combat forces promoting the need for major reductions in GHGs and a shift to renewable energy. This anti-reflexive DNA of climate denial serves to protect power and privilege systems formed since industrialization, which has been powered by fossil fuels.

## I. Introduction

Despite the consensus of over 99% of climate scientists that the Earth is warming primarily because of human-caused greenhouse gas (GHGs) emissions and that this warming has serious negative impacts [1–3], a recent national poll found that only a little over half (53%) of the American public believes that “most scientists think global warming is happening” while a quarter (25%) believe that “there is a lot of disagreement among scientists” on whether it is happening [4].

To a large extent, this misunderstanding reflects the impact of an organized climate change countermovement (CCCM) centered in the US which has cast doubt on anthropogenic global warming (AGW) and climate science among the public and policymakers for over three decades [5]. The CCCM was launched by fossil fuel corporations and other corporate actors dependent on fossil fuel use to protect their interests and was quickly joined by the US Conservative Movement well established in the literature [6–8]. This effort has evolved over time from mainstream conservative efforts (e.g., protecting markets from regulation) to now include more far-right programs. In the US this started with the Tea Party movement [9]. The literature indicates that the climate denial connection to the far-right include white power (whether stated or implied) [10,11], authoritarian tendencies [12] as well as specific far-right alliances to the fossil fuel industry [13] of contemporary climate denial [14,15].

Ekberg writes that climate denial has “clear signs of alliance with far-right actors” [16]. Literature characterizing the “far-right” [17], argues that the label “conservative” underappreciates the AGW denial is part of the CCCM’s reactionary effort to protect the status quo against progressive interests calling for change, especially in American society and protect the socioeconomic system based on a free-market economy with its promise of endless growth and unlimited progress-- to defend the “Western way of life” writ large [18–20]. It is increasingly recognized that the roots of AGW denial thus go much deeper than protecting the fossil fuel industry, which has literally fueled economic growth in the U.S. and much of the rest of the world for well over a century [21–23]. Yet, analyses of the claims used by the CCCM have not yet sufficiently verified these speculations.

There have been a number of empirical analyses of the claims issued by the CCCM over the past quarter century, and they have become increasingly sophisticated in terms of the collection and analyses of ever larger bodies of data [see especially [7,24–26]. We will review the most relevant studies shortly, but despite the insight they have provided into the nature and evolution of denial claims, we believe they fail to probe deeply enough to elucidate the fundamental, underlying rationale of AGW denial or what Lejano and Nero [23] label its “genetic narrative” or “DNA.” This paper reports our effort to achieve this goal by employing an in-depth qualitative content analysis (QCA) of a large number of books (108) espousing AGW denial [27]. We use a two-stage analytical approach, employing deductive and then inductive contextual thematic content analysis to read and code the books, yielding results that shed light on the underlying rationale for denial at a deeper level than currently exists in the literature.

Deductive content analysis is “directed” by existing understanding, and is used to “validate or extend conceptually a theoretical framework or theory” or, in the present case, findings from “prior research” [28]. We first use a deductive approach to intentionally discover and code denial of basic climate science--clarified early on by climate scientist Stefan Rahmstorf [29]--in the books: that the Earth is warming (trend denial), this warming is largely due to human-caused carbon emissions (attribution denial), and this warming is having negative impacts for humans and ecosystems (impact denial). We also code climate policy denial, involving a range of arguments against taking action to reduce carbon emissions that produce AGW. While AGW these four broad categories of denial claims and the numerous more specific claims within each one, have provided the foundation for questioning human-caused AGW and climate science for over three decades, and are commonly employed by Trump-era Republicans who often describe human-caused AGW a “hoax” [30]. These narratives have also been employed by CCCM organizations in several other nations as denial has spread internationally [31,32].

Typically, denial claims are embedded within larger narratives – what we term “semantic ecosystems.” A semantic ecosystem refers to the body of interconnected discourses of which the denial claim is one part. From a theoretical perspective, a semantic ecosystem is part of a discursive formation:

Whenever one can describe, between a number of statements, such a system of dispersion, whenever, between objects, types of statement, concepts, or thematic choices, one can define a regularity (an order, correlations, positions and functionings, transformations), we will say, for the sake of convenience, that we are dealing with a discursive formation… [33].

Krippendorff [34] describes this as a “semantic network”--binary relationships where “the meanings of nodes are a function of how they are connected with each other” (p. 292). The networks are detectable via <node_i_ – connection_j_ – node_k_> and contain “answers to questions that are not literally contained in a body of text but are implied by it” (p. 292). This approach is part of a theory of meaning which “originated in cultural-anthropological efforts to represent cognitive structures or mental models” (p. 293). Our nodes are connected by “proximities within a textual unit,” which are chapter sections in this case. We draw the connection between nodes through statements that directly and semantically tie one node to another. One node is always a statement of climate denial.

We analyze these ecosystems inductively, but in a more detailed and nuanced way than conventional inductive content analysis [28] which seeks to discover and systematize key claims from scratch, as employed by McCright and Dunlap (7). Deep examination of the semantic ecosystems around AGW denial claims leads to the discovery of two new, underlying meta-themes of denial on which the more specific denial claims (trend, attribution, impact and policy denial) ultimately rest.

These new meta-themes are full-fledged attacks on climate science (and increasingly on science at large) and global environmentalism in general, both of which are portrayed in the books as critical threats to capitalism, freedom, progress, and the American (and Western) “way of life” [see 19]. More studies of denial claims [7,15,16] have added a fifth major theme along with trend, attribution, impact and policy denial which challenges the integrity of climate science and scientists. However, we argue that this broad attack on science is in fact a deeper, more fundamental theme along with anti-environmentalism that underlies overall AGW denial. Our analysis shows that AGW denial stems from broader efforts to protect the sociopolitical and economic status quo, especially the power and privilege of those who benefit most from our fossil-fuel dependent economy.

Climate science is an “impact science,” a type of science that investigates the negative impacts of industrial production, and environmentalism can act as a reflexive movement critical of the structures of power that cause or facilitate environmental deterioration. Together, they represent a powerful challenge to the current market economy promoting endless growth. Opposition to this challenge represents “anti-reflexivity” [35,36].

We argue that fear of successful challenges to the status quo motivate AGW denial, and our analysis shows that anti-reflexivity--especially attacking climate science (and other impact sciences) *and* environmentalism (from local to global)--is its foundation. Our results suggest that anti-reflexivity is the underlying foundation or “DNA” of AGW denial.

## II. The climate change countermovement

A social movement is a conscious collective effort to organize for social change [37]. If a movement threatens to be successful, elites often will defend their interests and organize against the social movement in a “countermovement” [38,39]. As the global environmental movement threatened to successfully convince the international community to fight AGW thereby threating the political-economic status quo, elites organized an opposition project, a countermovement against the global environmental movement. This is the CCCM which started in 1992 after the Rio “Earth” Summit announced global environmental problems at the same time far-right activists were searching for an agenda after the collapse of the Soviet Union in 1991 [19].

The CCCM is well organized and effective, especially in publicizing its denial claims among the public and policymakers [40]. Early work showed several conservative fossil fuel-funded think tanks (CTTs) focused on AGW in the 1990s, portraying it as a “non-problem,” and that their efforts helped fossil fuel and other corporate actors defeat the Kyoto Protocol in the US [6–8].

These disinformation tactics became increasingly successful. When the Conference of Parties met in Copenhagen in 2009 hoping to replace the Kyoto Protocol with an equally ambitious institution. The so-called “Hopenhagen” meeting was a,

…pivotal moment in history when nationalist and corporate forces formed new alliances and sought novel ways to undermine climate resolutions. The denial machine’s ability to hinder cooperative and coordinated efforts between nation-states was demonstrated through an unprecedented attack on the summit using media and social media platforms to disseminate disinformation and fuel denialism [41].

Further, Brulle [42] reported that 91 countermovement organizations (including industry associations like the American Petroleum Institute and advocacy groups like Americans for Balanced Energy Choices, as well as CTTs) had a total income of over $7 billion between 2003 and 2010, providing ample resources for promoting AGW denial. Shortly after, Farrell [43] identified 164 organizations and 4556 individuals comprising the CCCM, providing insight into the networks linking them and how the actors and networks have evolved from 1993 to 2013. Lastly, while the US was the birthplace of AGW denial, it has gradually diffused to other nations, especially those with a history of economic dependence on fossil fuels [5], but to many others as well--typically through CTTs as a growing body of evidence suggests [27,31,44]. Still, the Anglo group of countries, “… the wealthy, English-speaking, primarily Protestant former British colonies with institutionalized white power: Australia, Canada, New Zealand, South Africa, the United Kingdom, and the United States…,” remain the bastions of AGW denial (although New Zealand and South Africa less so than the others) [45, p. 336].

As these examples illustrate, empirical studies are providing a growing body of evidence concerning the evolution, structure and impacts of the CCCM, as apparent from several reviews [5,46,47]. Here we will focus specifically on the growing body of studies analyzing AGW denial claims.

### A. Common claims/arguments used in climate change denial

Research on AGW denial claims and arguments has expanded considerably since McCright and Dunlap’s [7] early analysis of a sample of 224 documents on AGW posted by fourteen CTTs from 1990 to 1997, the year that over 160 nations met in Kyoto to develop the Kyoto Protocol designed to reduce Greenhouse gas (GHG) emissions. They employed inductive QCA to discover the “counterclaims” (to the claims made by mainstream climate scientists and concerned policy makers) about AGW, and discovered they fell into three main categories reflecting trend, impact and policy denial--each of which had a number of sub-categories [7, p. 510]. The main claims and common examples of each are: (1) “The evidentiary basis of global warming is weak and even wrong” (e.g., “The scientific evidence for global warming is highly uncertain”), (2) “Global warming would be beneficial if it were to occur” (e.g., “Global warming would improve our quality of life”), and (3) “Global warming policies [designed to reduce GHG emissions] would do more harm than good” (e.g., “Proposed action would harm the national economy”).

During this early stage of denial (1990–1997), there was so much emphasis on trend denial that attribution denial had not yet emerged as a distinct counterclaim. But as apparent from the Second and Third IPCC reports (in 1996 and 2001, respectively), scientific evidence for human contributions to global warming rapidly grew in the late 1990s, making attribution denial more common as Rahmstorf [29] noted based on arguments he often encountered as a prominent climate scientist.

Boussalis and Coan [15] reported a major expansion and update of McCright’s and Dunlap’s analysis [7]). They collected over 16,000 documents from 19 North American CTTs from 1998–2013, and then used unsupervised computational modelling to machine code key themes and empirically identify topics in this data. Over this period, they found that CTTs increased their production of climate denial documents “exponentially” (p. 97). They also found, as had prior analyses of denial books [27] and of conservative columnists’ op-eds [48], that the production of denial misinformation was not steady or random but responded to external events like pending AGW legislation. Boussalis and Coan identified 47 topics that they classified as dealing with either “science” or “politics and policy” and found they fell into five topic clusters consisting of “policy & regulation,” “domestic and international politics” and “energy and emissions” (politics and policy domain) and “climate science” and “scientific integrity” (science domain) [24, p. 93]. Furthermore, the pattern among the clusters mapped onto a two-dimensional space made sense, with the three policy-relevant clusters being close to one another but distant from the science cluster while the scientific integrity cluster is as close to the domestic and international politics cluster as it is to the science cluster–suggesting the political nature of questioning the integrity of climate scientists and institutions.

Scientific integrity was a newer theme that became especially prominent with the “Climategate Controversy” in 2009–2010, and Boussalis and Coan [24] describe it this way:

Not only do topics dealing with scientific misconduct—both regarding scientists themselves, the scientific consensus on AGW, and the IPCC in general—form their own distinct cluster, the language used seems to have more in common with politics than science; that is, scientists are presumed to wield ‘junk science’ to achieve political aims” (94-95).

More generally, they report that despite claims by some commentators that AGW doubt was declining, the levels of doubt about both climate science and policy expressed by the CTTs remained strong throughout the 16 years they examined. Finally, while their sophisticated methodology allows for the analysis of denial claims in huge bodies of data, Bousallis and Coan conclude by noting that, “… more detailed qualitative research is necessary to determine the prevalence of both new and past claims” (p. 98), which is exactly the lacuna we work to fill.

Farrell [49] conducted an even more comprehensive analysis focusing on 40,785 texts produced by the above-mentioned 164 AGW countermovement organizations including far right foundations, industry trade associations, lobbying firms as well as CTTs covering twenty years (1993–2013). Besides using large-scale computational text analysis to uncover major topics emphasized in this huge corpus, he also examined the role of corporate funding on the amount and content of the texts and the degree to which their content made its way into major newspapers, Presidential addresses, and Congressional proceedings (with most success with newspapers and Presidential communications). Of most relevance to the present study, Farrell’s [49] structural topic modeling methodology produced a 30-topic solution and further analysis showed formed four clusters he described as “questions about scientific evidence” for global warming, public knowledge and media (reflecting limited public understanding of climate science), federal and state-level politics, and the economic and political costs of enacting AGW policy (pp. 94–95). The 30 topics include examples of the early four key denial claims as well as suspicion of the IPCC and scientific authority (representative of the newer emphasis on scientific integrity), primarily within the large “scientific evidence” cluster.

For our purposes, the key outcomes of both the Bousallis and Coan (15) and Farrell (34) studies is their evidence that the original denial emphasis on trend, attribution, impact and policy denial remain prominent but continually evolve in response to new evidence and policy proposals, while the newer emphasis on climate science/scientists’ integrity (stimulated by the controversies over Al Gore’s and the IPCC’s Nobel Prize, Mann’s hockey stick model and the related “Climategate” affair created by the CCCM) has emerged as a major denial theme. The third and most recent major study reinforces these points.

Coan, Bousallis, Cook and Nanko [25] built directly on the detailed catalog of denial claims posted on “Skeptical Science” (a website run by Cook for several years), and prior studies of denial claims, especially [24], with their ambitious study of the claims issued by 20 prominent CTTs in the USA and Canada along with the claims posted on 33 “prominent climate contrarian blogs” (a majority from the USA but over a third from seven other nations) from 1998 to 2020—yielding 255,499 documents. Coan and colleagues carefully developed and refined a detailed taxonomy of what they term “climate contrarian claims,” involving the five “super-claims” reflecting the themes found in past studies: (1) “global warming is not happening,” (2) “human-caused Greenhouse Gases are not causing global warming,” (3) “Climate change impacts are not bad,” (4) “Climate solutions won’t work” and (5) “Climate movement/science is unreliable”—clearly reflecting trend, attribution, impact and policy denial along with broader challenges to the evidence put forth by climate science and those promoting efforts to reduce carbon emissions. They also developed “sub-claims” for many of the major ones and “sub-sub-claims” for most of the former. They then employed a supervised learning approach to classify the themes, first using a “team of climate-literate coders” to code a huge sample of paragraphs into the typology and refining the latter when necessary, and then trained a computational model to classify claims in their corpus into the taxonomy. They also added in data on the major funders of the CTTs and key legislative and societal events.

Their impressive data set and methodology enabled Coan, Bousallis, Cook and Nanko [25] to provide a rich analysis of denial claims over time and the factors that influenced variation within and among them. Of utmost importance is that their taxonomy of claims was validated, with all five being used over the 22 years studied but with varying frequencies. Crucially, they found growing attention to policy denial (“solutions won’t work”) over time—especially from the CTTs. They also found high but slightly declining levels of “science is unreliable” from both CTTs and blogs. The three categories of claims reflecting trend, attribution and impact denial have been stable or slightly declining for the CTTs, but remain stronger for the blogs—particularly for trend and attribution denial [25, Fig 2]. According to the authors, “these results suggest that the blogs seem to be acting as the pseudo-scientific arm of the AGW counter-movement” (Ibid., p. 4).

**Fig 1:**
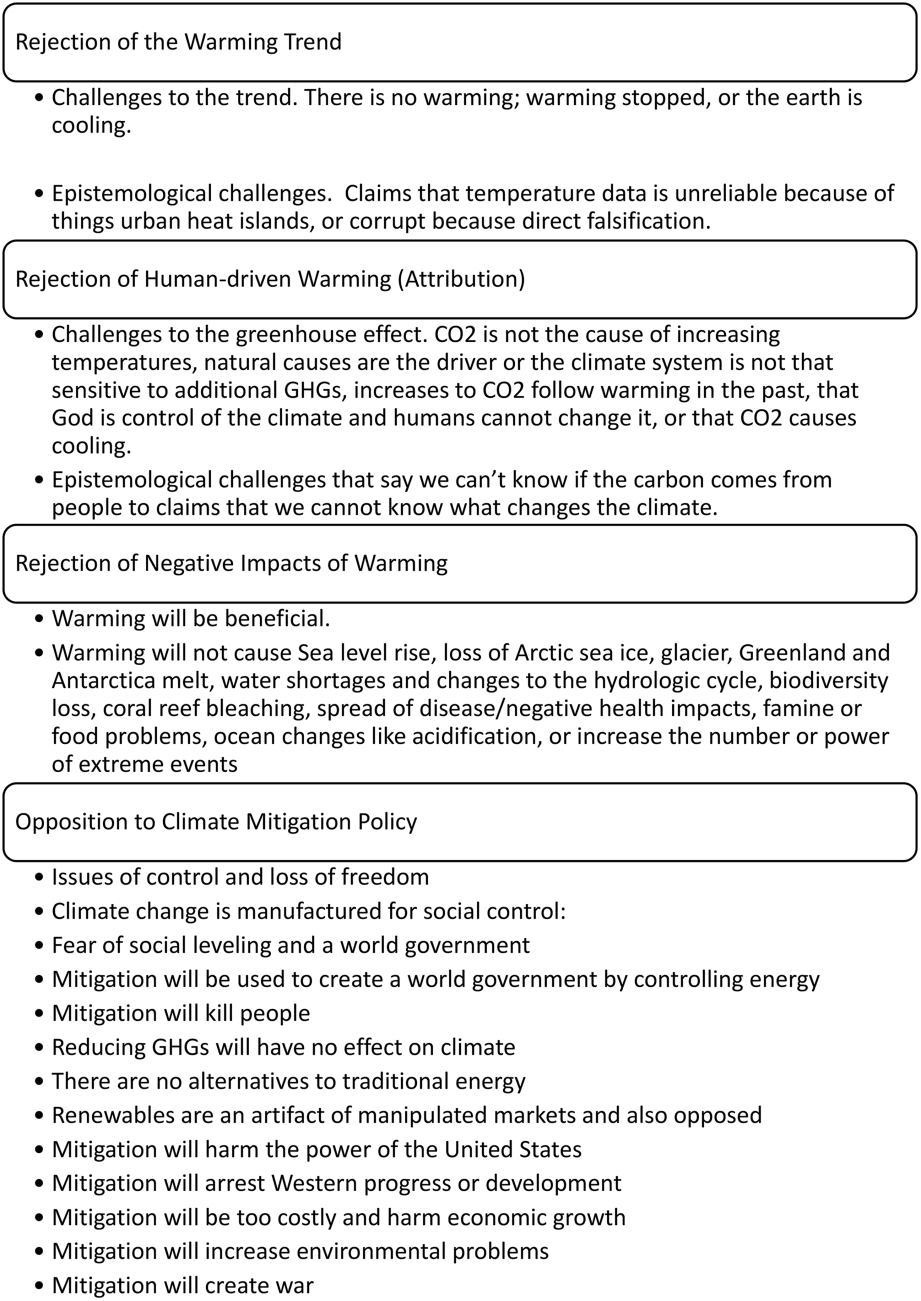
Summary of Climate Denial Claims.

**Fig 2:**
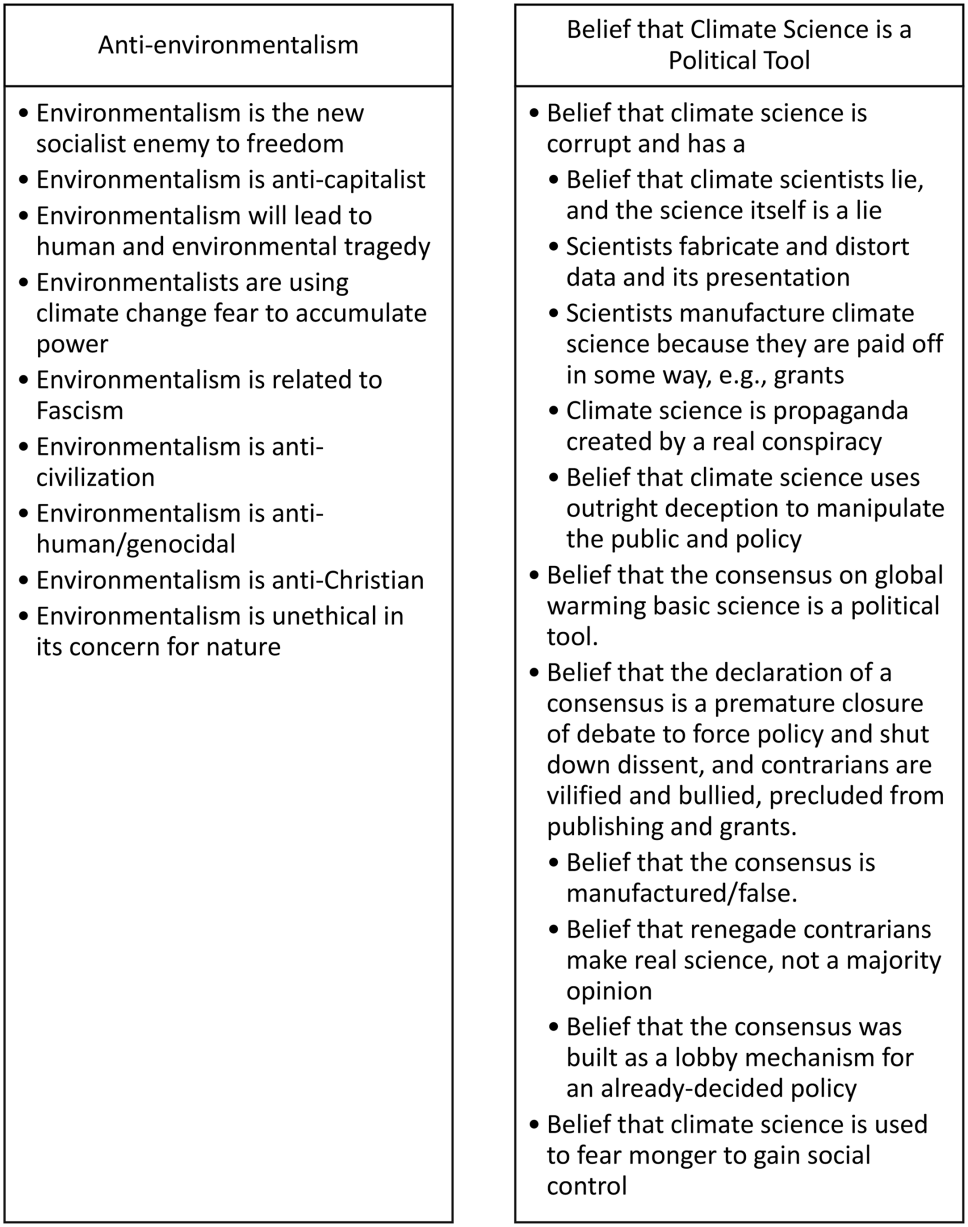
Anti-Reflexivity in Climate Denial Books.

The three studies just reviewed, using huge samples of documents produced by major elements of the CCCM, covering long time periods and employing sophisticated analytical techniques, have clearly shown that while denial claims evolve over time in response to advances in climate science and socio-political events, the four original denial claims (mainly from the 1990s and early 2000s) have never disappeared but have been supplemented with newer ones (especially scientific integrity). Further, several other studies using somewhat more limited data sets (in terms of their corpora), but covering a wider variety of sources and nations than the above three, have overall yielded compatible results: the continuing use of trend, attribution and impact denial, the growing importance of policy denial and of challenging the integrity of climate scientists, climate science, the IPCC, climate policy-makers and activists [31,44,50–52]. As a true “countermovement,” the CCCM constantly reacts to the claims issued by climate scientists and policy-makers and modifies its counterclaims in response.

The empirical evidence on the nature and evolution of AGW denial claims has rapidly grown and matured, but Lejano and Dodge [22] argue that “Future work involves going deeper into the roots of the climate skeptical narrative, perhaps uncovering a more elemental, genetic narrative on which it is founded” (p. 212)—precisely the goal of reading and coding the 108 climate denial books under examination. These books provide more in-depth presentations of denial than typical CTT reports or news releases. They also convey substantial legitimacy, and often receive considerable visibility—giving their authors credibility with the media, the public, and some policy-makers [27]. They therefore offer the opportunity to gain critical insights into the CCCM’s relentless efforts to question the seriousness of AGW and thus the need to reduce carbon emissions.

### B. Anti-reflexivity

Individual denial is informed by elite cues [53]. These cues are produced by “the US conservative movement (backed by much of corporate America) [which] seeks to delegitimate and defund impact science, continually pushing for cuts in the budgets of the Environmental Protection Agency and other agencies and challenging their regulatory authority” [36, p. 2].

Surveys indicate that political orientation has long been the best predictor of views of AGW, with Republicans and conservatives most likely to hold doubtful views including outright denial that it is occurring [4,54–56]. Conservative white males are especially likely to hold doubtful views, consistent with the predominantly conservative, white male constituency of the CCCM [57].

AGW threatens the far-right because it implies the need for deep socio-economic change and a redistribution of social power. Thus, the “anti-reflexivity” thesis was proposed by McCright and Dunlap [35] to explain AGW denial [see also [36,58,59]. Anti-reflexivity is the combined rejection of Reflexive Ecological Modernity *and* “impact science.” Reflexive ecological modernity calls for ecological rationality in governance and economics [60]. Reflexive modernity comes from Ulrich Beck’s [61,62] Risk Society thesis that calls for progressive cross-national citizen efforts, like the global environmental movement, to temper powerful forces that create planetary crises. “If such [efforts] are accompanied by reflection, openness to alternative understandings, and critical questioning, then we can speak of *reflexive modernization*” [63].” But if they provoke “… angry rejection of alternatives and retreat into the familiar by people who now understand the nature of the threat to them, we can speak of *reflexive traditionalization*” (Ibid. [emphasis added]). Thus, reflexive modernization considers alternatives to traditionally hegemonic ideas, but this threatens those empowered by that traditionalism, who then resort to anti-reflexive actions and discourses [35].

Earlier Schnaiberg [64] distinguished between “production science” that facilitates technological advances fueling industrial capitalism, and “impact science” that investigates the resulting negative effects of production and growth on humans and the environment. Along with reflexive modernity, impact science shows that the hazards and risks of industrial society “shake the foundation of industrial society, including its core institutions (e.g., the nation-state, science, systems of welfare and insurance)” [65]. Accordingly, McCright, Dentzman [66] show that conservatives distrust impact science but trust production science, likely because conservatives are prone to system protection and system justification [67,68].

One important point in the literature is that neither science nor international civil society are automatically or always reflexive. Drawing on Bowden, Nyberg, and Wright [69], Boström and colleagues note that some actors have the capacity of reflexivity and use it, “as a means to defend one’s privileged position” [65], purposefully creating and distributing ignorance. Thus,

…as well as being a tool for change, reflexivity is also a target of anti-reflexivity forces that aim to prevent change. It is therefore also crucial to address the powerful forces that deliberately and strategically counteract reflexivity, which many times are the same forces that create environmental destruction [70].

In sum, anti-reflexivity opposes ecological reforms, progressive social movements, and science that provide critiques of capitalism. Anti-reflexivity is likely an ontological and epistemological funnel shaping being and knowing contingent on inexpensive hydrocarbons and which reorients, “those with relative privilege as ‘victims’” [69]. Anti-reflexivity’s effectiveness is enhanced because it is employed by powerful institutions to combat challenges to the status quo [35].

## III. Materials and methods

In this study, we use QCA, which Krippendorff [34] describes as, “one of the most important research techniques in the social sciences,” although it does have some inherent limitations—e.g., theory built from the material analyzed cannot be generalized. However, QCA reveals “the symbolic richness and uniqueness of the data at hand” (Ibid, p. 407) which we can use to develop a theory about the “genetic narrative” for climate denial.

Berelson [71] notes that qualitative work is normally done on small, often incomplete, samples to understand the “*’reflection’ of ‘deeper phenomena’*” (p. 123, emphasis in original). The focus is on, “simple reading plus interpretation in the traditional sense—i.e., reading plus a judgment as to what the content ‘means.’” [71, p. 128]. Naturally, QCA requires a deep familiarity with the content, as such, we have devoted an enormous amount of time immersing ourselves in close readings of the large number of denial books in our data set.

Schreier [72] notes that qualitative content analysis has three main elements: it reduces data, it is systematic, and it is flexible. In reducing data, the research focuses on select material. In being systematic, coding is done through the same steps, such as processing coding frames. The flexibility of QCA means that as the text is understood, the coding frames (can) change to match the material in a data-driven fashion.

The coding frame, or the way coding is organized, is a critical element of QCA. Each part of the frame should cover one area and be mutually exclusive to other major frames. Following Schreier [72], our coding frame is constructed through two questions: (1) “what denial claim” and (2) “why.” Thus, the coding frame covers the assertion and the rationale.

### A. QCA stage one: identifying denial

Systematically understanding the content of over 100 books presents a challenge. Our approach to reducing data relied on theory that followed first deductive then inductive logic, akin to the thinking of George E.P. Box’s [73] explanation that scientific learning follows an progressive and iterative dynamic between deductive and inductive advances.

These English-language books were identified through bibliographies of known climate denial writers in both their books and articles, online blogs that promote climate denial, academic publications about climate denial, and online searches. A “book” is defined as a publication with an International Standardized Book Number (ISBN) and books were chosen for rejecting climate science to create a comprehensive database in the time period. The database is significant because it represents a robust sample of the climate denial literature, although some books are more important/influential than others. Some are written by people with advanced degrees, e.g., Patrick Micheals who has a Ph.D. in climate science and whose books are promoted by think tanks like the Cato Institute. Others are self-published books by authors with little expertise connected to AGW. Importantly, as noted above, the study is limited to books published by 2010. Also as noted, studies show that the rationale for denying AGW has evolved since 2010, yet the core elements (trend, attribution, impact and policy denial) have remained but are updated in response to new scientific evidence and relevant societal events. For example, analyzing Heartland Institute documents from April 2014 to June 2015, Cann and Raymond [51] find that science frames continue to dominate these texts, but with a “greater reliance on *ad hominem* attacks on climate scientists and their supporters” (p. 444), a good example of the “scientific integrity” theme that has become more prominent over time [24,25]. Nonetheless, attacks on the integrity of climate scientists can be found in some of our books, the earliest being Daly (1989) who called “qualified scientists” “charlatans” and “armageddenists” who used fear mongering to cower the public (p. 172–173). Note that references to books like Daly’s that are part of our dataset are listed in the SI, *not* in the references, and are not numbered but identified parenthetically by author and date in the text to distinguish them.

Deductive learning tests theory, and inductive learning builds theory. Since the denial of AGW *science* is defined by trend, attribution, and impact denial, *each book must contain at least one of the these three denial claims* to be included in the dataset [29]. We also identified policy denial as a key claim made by CTTs early on [7], but it was *not* a requirement for inclusion. However, given the prominence of policy denial, stage one of the analysis uses all four categories to identify denial claims.

Our analysis began with one of the authors reading all of the books three times, marking potential passages that reflected one of the four key claims of AGW denial. Then, a team of eight climate literate undergraduate researchers and the same author coded the passages for one or more of these claims in reading and discussion sessions where there had to be agreement on each code.

One of the main challenges to deductive content analysis is inter-subjective validity [74]. Rourke and Anderson [75] argue that the process for developing valid protocols involves identifying the purpose and the behaviors/concepts that represent the construct (categories), reviewing these categories, testing them, and developing guidelines for interpretation. To determine if the coding rules were reliable, a second set of eight undergraduate researchers and an author coded 13 passages with basic rules for either a rejection of a warming trend, humans’ contribution to GHG emissions, that warming will have negative impacts, and the need for policies to reduce GHGs for a total of 117 decisions with 468 potential possibilities. Under these rules the inter-coder reliability was near perfect between the nine coders (Cohen’s Kappa,.892; Scot’s pi,.891), indicating our rules were reliable. Then, in order to analyze passages in MaxQDA 11.1.2 software (Stage Two), we digitized relevant coded passages into portable document files (pdfs), paying attention to the semantic ecosystem.

It is important to note that climate denial authors are not always consistent in their claims or rationales, and it has already been noted that incoherent claims of the “world is cooling” can coexist with claims that warming is “natural” [76]. For example, Michaels (2004) proclaims, “Global warming is real, and human beings have something to do with it” (p. 9) but then in the same book warns that, “…IPCC’s scenarios of dramatic warming require the continuation of an exponential increase in carbon dioxide that stopped a quarter-century ago….” We solve this problem with data-driven empiricism by coding the plain language rejection of climate science where it occurs in the text, or “manifest” coding. Coding of a book stopped if all four denial categories were found--thus our data do not include every denial claim in every book.

### B. Stage two: open coding technique

After the denial code, we move to unrestricted, inductive open coding of the rationale for denial. Open coding, sometimes called emergent coding, means the rationale for denial emerges directly from the data allowing for theory building to occur as a result. As Cope [77] indicates, “The purpose at this stage is to ‘open up’ the data,…so that conceptual implications can emerge…” (p. 446). Typically, the narrative under analysis was one or two pages proximate to the denial claim within a chapter section. The length of the text unit was determined by the semantic relations which are “instantiated by context” of chapter sections which usually address a focused aspect of the narrative arc [78].

Each semantic network is in the form of speech acts by a discourse community and over time patterns emerge. As the patterns emerge, so does “coherent discourse” – i.e., the argument is legible [79]. In our data, we identify “cause and effect” as the coherent relation, which Hume [80] described as one of “three principles of connection among ideas” (p. 32)- the other two being “Resemblance, Contiguity in time or place” [67]. Coherent cause and effect discourse infers: “P from the assertion of S_1_ and Q from the assertion of S_2_, where normally P → Q” [79]. In other words, we can infer how one assertion must plausibly follow another with or without plain exposition. If P and Q exist within proximate passages, the discourses co-occur and co-occurring discourses inform each other’s meaning. We assume that words used in each book are related, and it is important to understand how the discourses are associated in the spirit of linguist J.R. Firth [81] who noted that we understand the meaning of words by the “company it keeps.” So, when one claim is in proximity to another claim, together they create larger meta-discourses because the denial claim does not exist alone but within a forest of meaning that comes from the surrounding narrative. This is part of latent semantic analysis:

LSA assumes that the mind or brain applies essentially the same method to mapping the meaning of words (and other experiences) into semantic space. It starts with rough estimates of closeness in the form of local temporal associations, then finds a way to fit them together [82].

Take for example, a denial code from stage one from a section by Glover and Economides (2010) in a chapter called “The Pseudo-science War on Carbon and Climate:”

But the global mean temperature has *not* continued to rise. The natural explanation is that CO2 emissions are *not* the causal factor when temperature rises. But, once again, the

“natural” explanation is not acceptable to AGW-theorists. Too many science grants and personal reputations are now staked on high-profile alarmist predictions. The reality is that CO2 emissions are continuing to rise around the world while the global mean temperature has remained static for over a decade, and may even have fallen. (p. 98, emphasis in original).

Then reading around this quote in stage two coding in the subsequent paragraph, are attacks on the integrity of a science where scientist are accused of ignoring facts to advance an environmentalism that is “anti-human” and a dogmatic religion:

That should be enough to ‘hole’ AGW-theory below the waterline. And if we were dealing with real science it would, and the vilification of CO2 would be at an end (or at least suspended pending further investigation). *But we are not dealing*

*with pure science.* We are rather dealing with science suffused with a strong, anti-human, eco-faith agenda. Facts and data are ultimately, for AGW activists~ non-essential elements of their faith (Ibid).

Thus, the denial claim is narratively related to the paragraph after it (together, the textual unit), using the indicator “That should…” to indicate a successive point and the semantic connection between nodes articulated directly in the text. The codes are related to each other through the coding frame of “what denial” and “why denial.” This interrelatedness is usually observed in stage one but not analyzed until stage two reading and coding. Through this process, we are able to analyze the larger narrative beyond the simple denial claim to get at the narrative’s “DNA.”

Another example illustrates anti-environmentalism in stage one coding identifying impact and policy denial:

Second, and more important, we know by now that a warmer climate is a better one, most certainly during the early stages which we might observe during the next decade. Therefore, the most likely outcome of an ongoing trace-gas build-up is not a catastrophic event but a beneficial one. Taking out insurance and paying premiums against coming changes for the better seems to be the height of folly… Luckily we know that there is not going to be any damage to prepare for at this time (Weber 1991, p. 136-137).

The following paragraph connects the denial claim to the larger rationale for denial discovered in stage two coding where the author directly and semantically connects the denial claim to a larger rationale, “But now we come to an additional point of overriding importance” which are the “ulterior motives” and “ the plans of some of the *environmentalists*, who are *against* anything that has to do with industrialization and comfort provided by technological *progress*” (Weber 1991, p. 137, emphasis added). This passage is written proximately to the above denial claims to explain the reason for the denial claims—AGW science is unsound but that does not matter to environmentalists who advance the issue *because* AGW fits the environmentalists political agenda:

These groups are obviously not very interested in questioning the scientific basis of the global warming/ greenhouse hypothesis, because that would be self-defeating to advancing their ulterior goals, so in order to add weight to their demands, they make the whole matter look as bleak as possible, regardless of the scientific soundness (p. 137-8).

In both examples, the denial claim and the reason for denial are in the same discussion in the chapter section and are tied to each other through direct explanation. These samples demonstrate the insight provided by examining the larger semantic ecosystem, and the opportunity to read more deeply into denial narratives, *which we would miss if we looked at the denial claim alone*.

#### 4.1 Results of stage one coding.

Table 1 shows the frequencies of the denial codes from Stage One, which indicates most books (94%) reject GHGs attribution and mitigation policy. All books had to make a claim in at least one denial category to be included in the population, but the average book made claims in 3.3 categories. Thus, a majority of the books rejected most climate science categories and the need for GHG-reduction policies. In fact, about half of the books (49%) expressed all four categories of denial and 77% expressed at least three kinds of denial. However, only *three* books kept to one category of science rejection and therefore did not make a case against mitigation policy, demonstrating the rarity of this position. This tells us there is a high degree of consistency and durability amongst the denial claims across nearly thirty years.

**Table 1:** Books that Assert Attribution, Trend, Impact, and Policy Denial.

	Trend Denial	Attribution Denial	Impact Denial	Policy Denial
**Total**	87	101	82	102
**Percent**	81	94	76	94

Here we describe some of the findings, quoting sparingly for space, from the data listed in the supporting information, again, cited with parenthetical references to distinguish them from academic references in the work cited list.

#### 4.2 Rejection of the warming trend.

Trend denial occurs in three major claims: warming never occurred, warming stopped, or that the data describing warming is unreliable. Here are representative claims made by authors in the dataset, with each bullet point a sub-theme of the larger denial category:

- That there is no warming at all

“They show that the greenhouse heat is entirely dissipated to outer space by the cloud generated by the greenhouse effect. Therefore there is no greenhouse heat left on earth to generate global warming” (Fong 2005, xv).

- That warming stopped from somewhere between the 1970s to the 2000s:

There is irrefutable scientific evidence that global temperatures are NOT rising (in fact, temperatures have dropped slightly since 1998).…” (Huseman 2010, back cover).

- That temperature data is unreliable, e.g., land and satellite temperature measures do not agree, or that urban heat islands corrupt temperature measurements, and that climate scientists cannot be trusted, e.g., Mann’s “hockey stick” temperature reconstruction.

According to the USHCN [US Historical Climatology Network run by National Oceanic and Atmospheric Administration] archives, the temperature has warmed only.5°F since the mid- l800s. Tossing out corrupted USHCN data, there has actually been a net-cooling since 1930-during the same period in which atmospheric C02 has noticeably increased (Sussman 2010, p. 61).

### 4.3 Attribution denial

- Claims span from “natural causes,” e.g., increasing solar heat, to a climate system insensitive to additional carbon, to claims that the carbon dioxide does not stem from human emissions, to claims that we cannot know what causes changes in climate. Authors often call climate models unreliable, such as Cotton and Pielke, Sr. (1995), while others simply cast doubt on the entire greenhouse theory in general. Cotton and Pielke, Sr.’s first edition was self-published by Aster* Press, then Cambridge University Press published subsequent editions in 1995 and 2007. Assuming CUP had it reviewed, this appears to be the only peer-reviewed book in the data (making 99% of the denial books unvetted by peer-review), which may be why the language is much more tempered and careful compared to other books.

An example of this type of claim is found in Macrae:

From the start, instead of claiming “certainty,” a concept that has no place in science, climate scientists could have explained to the public that their models are tentative. They could have admitted in 2007, when the non-warming of the 21^st^ century became clear, that the planet wasn’t warming, rather than attacking those who were simply stating the truth. lnstead, the IPCC’s 2007 report declared that warming of the 21st century was “unequivocal,” an astonishing conclusion but quite in line with the IPCC’s agenda of creating climate fear rather than climate reason (Macrae 2010, p. 310).

- The role of CO2 in AGW is exaggerated or wrong.

The greenhouse effect is considered to be fact, whereas the enhanced greenhouse effect is an unproven theory (Bate and Morris, 1994, p. 14).

And

When viewed in geological context, and assessed against factual data, the greenhouse hypothesis fails (Carter, 2010, p. 29).

The earliest book in the data set, *Carbon Dioxide: Friend of Foe?* (Idso 1982), claims that carbon actually cools the climate:

…what conclusion could possibly be drawn from these global temperature data other than that enhanced atmospheric CO2 concentrations either tend to *cool* the Earth or to exert no climatic influence whatever? No one who is truthful to himself can honestly say otherwise (p. 59, emphasis in original).

- Doubt that increasing CO2 is from human emissions.
  - Only God can change the climate.

#### 4.4 Impact denial.

Impact denial claims argue that increased warming/CO2 will be beneficial or rejects negative impacts.

- A warmer world will be an improved world.
  - A rejection of a long list of problems:
  1. Sea level rise
  2. Loss of Arctic Sea ice
  3. Glacier, Greenland and Antarctica melting
  4. Water shortages and changes to the hydrologic cycle
  5. Biodiversity loss
  6. Coral Reef bleaching
  7. Spread of disease/negative health impacts
  8. Famine or food problems
  9. Ocean changes like acidification
  10. Increasing number or power of extreme events

For example

One of the most feared consequences of global warming is a rise in sea level that could flood low-lying areas and damage the economy of coastal nations. But actual evidence suggests just the opposite: a modest warming will reduce somewhat the steady rise of sea level, which has been ongoing since the end of the last Ice Age--and will continue no matter what we do as long as the millennia-old melting of Antarctic ice continues (Singer 2000, p. VI).

Likewise, Jastrow, Nierenberg, and Seitz, founders of the George C. Marshall Institute which was central to the early CCCM [83], write that:

Warming leads to lower sea levels because warming will increase precipitation over Antarctica and Greenland (Jastrow et al., p. 159, emphasis added).

#### 4.5 Policy denial.

Almost all of these books oppose climate policies designed to reduce carbon emissions, and we highlight several claims below. Note that as social concerns, the results of this coding form a bridge to some narratives found in Stage 2. There are many arguments provided against mitigation policy:

- Fear of loss of control and freedom

Environmentalism validates intrusive regulation, punitive massive bureaucracy, and even world government...We in the industrial world must make massive sacrifices-particularly in America (Murray 2008, p. 3).

- Climate change is manufactured for social control.
- Fear of social leveling where mitigation policy will be used to create a world government by controlling energy.
- Mitigation policy will kill people.
- Reducing GHGs will have no effect on climate.
- There are no alternatives to conventional energy.
- Mitigation policy will harm the power of the United States.
- Mitigation policy will arrest Western progress or modern development.
- Mitigation policy will be too costly and harm economic growth, particularly in the US, again in nationalist tones:

Any other legislated measures will constitute an assault on the liberty and economic well-being of the American people and a disruptive exercise in futility. When the science of global warming and AGW can no longer be ignored, suppressed or censored, the promoters of the man-made global warming hoax will no longer be able to impose an authoritarian agenda through fear and hysteria (Johnson, 2008, p. 66).

- Mitigation policy will increase environmental problems.
- Mitigation policy will create war.

The above results confirm the heavy prevalence of the four key denial claims documented thus far in the literature. The claims are summarized in Fig 1. Note that some of these fears, like a fear that mitigation will arrest Western civilization progress, mimics fascist ideology and far-right nationalist views found in the Belle Époque (late 1900s) by the ultranationalist futurist movement in pre-WWI Europe [84,85].

### 5.1. Results of stage two coding: the DNA of climate denial

Reading the corpus of books reveals a larger coherent discourse, signaled first by denial claims. The emergent coding reveals two fundamental logics: anti-environmentalism and a genuine distrust of climate science. These meta-themes operate like genetics, or the fundamental rationales, for climate denial. They provide, “a certain thematic [which] is capable of linking, and animating a group of discourses, like an organism with its own needs, its own internal force” [33].

### 5.2 Anti-environmentalism

Climate denial rhetoric expresses a deep anti-environmentalism, consistent with increasing polarization over AGW [4,86]. There is open animosity toward environmentalists, often affiliated with former Vice President Al Gore whose “Inconvenient Truth” film, book and Nobel Prize generated a lot of opposition from the CCCM. There is a general sense that environmentalists are socialists in disguise, working to achieve global power at the expense of individual freedom, capitalism, US power, and Western progress.

For example, Vaclav Klaus, former President of the Czech Republic, in a 2007 book published by the Competitive Enterprise Institute warns that reducing greenhouse gases will be disastrous for humankind/civilization/Western progress and will send civilization back to the Stone Age. The force that threatens to do this is environmentalism.

- Environmentalism is the new socialist enemy to freedom.
- Environmentalism is anti-capitalist. Horner (2007) in his chapter entitled, “Green Is the New Red, The Anti-American, Anti-Capitalist, And Anti-Human Agenda of Today’s Environmentalists” writes:

Spawned from the 1970s split of anti-modernists from the decades-old conservationist movement, “environmentalism” has matured into a nightmare for anyone who believes in private property, open markets, and limited government. Environmental pressure groups have no use for limiting governmental powers or expanding individual liberties. Instead, environmental claims are without fail invoked to advance the statist agenda (p. 3).

- The lies of environmentalism will lead to human and environmental tragedy and environmentalists have a secret, corrupt agenda.
- Environmentalists are simply using AGW to accumulate power.
- Environmentalism is akin to totalitarian fascism. This probably comes from resource nationalism, where hydrocarbons are fetishized and limits to using them endangers national identity [87].
- Environmentalism is opposed to modern society or civilization:

Environmentalism is anti-human.

Environmentalism is anti-Christian, an evil religion and is analogous to genocide:

Because they are not prepared to look at the truth of Jesus Christ, people are being deceived by falsehoods. Yet even the science they claim to believe in is telling them that they are accepting lies. When concern for the environment got out of control decades ago now, it rapidly assumed the status of religion. But it is a false one, perhaps *the* false one. “By their fruits you will know them” and what do we see? (Foster, 2009, 188, emphasis in original).

- Environmentalism is unethical in its concern for nature.

There is a “deep anthropocentric” ethic in the data consistent with anti-environmentalism, where humans are the only subject of value [see 88]. Roy Spencer, another key figure in the CCCM, argues for a deep anthropocentrism. This ethic informs what policy norms are acceptable, and posits environmentalism as an illegitimate religion:

In general, environmental policy decisions that favor nature over people are based upon worldviews or religious beliefs that are separate from the science…. Any rights that we confer upon nature through environmental policies should only be those that benefit humanity. Anything else verges on a state-supported Pagan religion (Spencer, 2008, p. 180).

This ethic also contends that changes to the environment are not negative if they improve human lives. The logic is that AGW has been inconsequential because “nature” has no value other than for humans, and for Spencer, an industrialized capitalist economy is far more important.

### 5.3 Distrust in the integrity of climate science

Authors argue that climate science—facilitated by media and government—is not reliable because it is a political tool created by a real conspiracy of scientists, co-opted by a radical, zealous, environmentalism that aims to exert religious-social control as noted above while at the same time about 99% of these books are published without peer-review.

There is a belief that climate science is deeply corrupt, and this corruption goes to the highest levels of the IPCC, the US government, and media.

- Climate scientists lie to the public.

Ralph Alexander wrote the 2009 book, *Global Warming False Alarm: The Bad Science Behind the United Nation’s Assertion that Man-Made CO*_*2*_ *Causes Global Warming*, and in his first chapter, entitled “Global Warming Deceit” writes:

The alarmists have spun such a web of deception that any science contrary to the view of human-induced global warming is either ignored, played down, or deliberately distorted (Alexander, 2009, p. 1-2).

Orthodox climate science is propaganda by a real conspiracy.

- The consensus on AGW is a political tool for social control and closure of debate.
  - Scientists manipulate data to scare the public for personal and professional profit.

Fig 2 summarizes the key beliefs that underlie the rejection of climate science in the two major elements of anti-reflexivity (anti-environmentalism and anti-impact science) as conceptualized by McCright and Dunlap [26].

### 6.1 Conclusion and discussion

We have used a two-stage analytical approach to examine over 100 climate denial books involving first a deductive then an inductive process. The goal was to understand the genetic narrative—the “DNA” of AGW denial at a level of detail heretofore unreported in the literature, providing new insights about denial, and the CCCM.

Using existing theory to identify climate denial claims in the books [29], we confirm the major denial claims reported in the literature. We find that while the two most frequent claims are attribution denial and policy denial, a majority of the books exhibit most of the types of AGW denial—even as they are modified somewhat in reaction to scientific and societal events. This indicates a robust consistency and durability of denial themes across more than three decades.

The most important findings are from our analysis of the semantic ecosystems in which these long-known categories of climate science and policy rejection exist where we discover new lines of reasoning, in particular strident anti-environmentalism. We find support for the findings of Boussalis and Coan [24] and other recent studies that AGW denial includes systematic attacks on the integrity of science and scientists, here framed as a branch of impact science. While Boussalis and Coan correctly note that this theme has grown stronger in the overall body of denial literature (reports, policy briefs, PR releases, etc.) used in their large computational studies, it was present in a few denial books from the beginning. We add to this an extreme opposition to environmentalism that has not been accounted for in the literature. Together, the opposition to the global environmental movement and attacks on impact science constitute the core elements of “anti-reflexivity” [35]. Anti-reflexivity is a powerful defensive mechanism employed by dominant interests to protect power and privilege ensconced in global capitalism fueled by cheap hydrocarbons and is consistent with far right nationalism, the protection of “petro-masculinity,” and white power. Dagget writes: “For many, extracting and burning fuel was a practice of white masculinity, and of American sovereignty, such that the explosive power of combustion could be crudely equated with virility” [15]. Likewise, Anshelm and Hultman [89] report, “These climate sceptics tried to save an industrial society of which they were a part by defending its values against ecomodern hegemony” (p. 84). Allowing meaningful restrictions to fossil fuels endangers the power of petro-privilege.

Hultman et. al. agree: “…far-right nationalism and AGW denialism [are] based upon the ideological similarities in viewing the world from an industrial masculinities viewpoint, not wanting to let go of the colonial extractive logic that has served these men well, but violated the planet” [90]. As Malm and the Zetkin Collective [14] show, following Griffin [91], far-right ideology, including fascism, has a historical relation with denial, lies, and misinformation since Mussolini. This helps us theoretically ground misinformation (not intended to deceive) and disinformation (intended to deceive)as forces that are capable of redistributing power and obscuring alternatives, “as when a traveler is falsely told a bridge collapsed” [92] notes Bok, and in this case our bridge is limiting fossil fuels to preserve a habitable planet.

Also, far-right ideology is palingenic, that is, obsessed with re-birth, and AGW denial can be a re-birth of that privilege that comes through the data with nostalgia for a time when white patriarchy was fueled with abundant fossil fuels. Under this lens, greatness and progress need to be defended and reclaimed from environmentalists and scientists. Anti-reflexivity provides a rationale to reclaim this “stolen pride” —something supporters of US President Donald Trump directly articulated to sociologist Arlie Russell Hochschild, speaking from the poorest county in the US while the president’s 2025 budget removed social safety-net essentials to pay for tax credits for the wealthy [93].

As conservative identity is forged around system protection and justification [67], attacking and delegitimizing AGW is expressed alongside a deep fear of losing essential conditions of the current dominant system, the neoliberal capitalist economy and white male dominance. Thus, it makes sense that the far-right joined the fossil fuel industry in organizing the CCCM, which in turn has forged a strong bond with the US Republican Party. While the GOP has adopted an increasingly anti-environmental record since the Reagan era, its opposition to environmental and especially AGW policies have escalated. The Bush 43 Administration, which was the primary focus of McCright’s and Dunlap’s [26] original analysis of anti-reflexivity, was extremely hostile to AGW policies but employed strategies designed to draw limited attention from the public. For example, it misrepresented and suppressed research highlighting the threats posed by AGW, it portrayed AGW policies as threatening our national well-being, promoted the work of a small number of contrarian climate scientists and pseudo-scientists, and supported attacks on the work of mainstream climate scientists in Congressional Hearings run by the GOP—sometimes resulting in vicious threats against the scientists [94].

Yet, the Trump Administration in less than two terms has exceeded all prior efforts to deny the reality and dangers of AGW by openly and aggressively attacking AGW policies, withdrawing the US from the Paris Agreement, and sabotaging international cooperation to reduce carbon emissions, putting AGW deniers like Scott Pruitt in charge of agencies such as the Environmental Protection agency in the first term, promoting energy production with no regard to environmental consequences, and much more [95]. And crucially, Trump normalized labeling AGW a “hoax” and a “scam” among Republican politicians, reflecting the success of the CCCM’s efforts to instill hardcore denial into one of our nation’s two major political parties (or at least what remains of the GOP under Trump). In July of 2025 during his second term, the Trump administration hired two authors in our data, John Christy and Roy Spencer, to the Energy Department. Immediately the Energy Department published a report authored by them and three others [96] questioning the validity of the “endangerment” ruling by the Environmental Protection Agency in 2009, twice affirmed by the US Supreme Court. Under the Clean Air Act, the EPA is authorized to regulate air pollutants that endanger the public, thus an endangerment ruling under the guidance of a scientific committee, is needed for the US to regulate CO_2_:

High impact weather extremes, usually related to temperature, precipitation and/or high wind, can disrupt infrastructure and therefore endanger human health and wellbeing. The issue is not whether extremes occur. Rather, it is whether there are long-term (decadal scale) changes in the frequency or character of extremes (“detection”), as well as the extent to which such changes and the attendant changes in hazards are caused by anthropogenic emissions of greenhouse gases… However, it is naïve to assume that any recent extreme event is caused by human influences on the climate (p. 46).

The EPA used this report to justify revoking the endangerment ruling and removing the authority for it to regulate and mitigate climate change. The policy process is ongoing as of this writing but the power of climate denial and the CCCM are obviously important.

All of this speaks to an agenda to defend the structures of Western, white power from a global problem many believe cannot be solved if that very system remains in place [97,98]. Indeed, these far-right moves have led the Polity project, which rates countries for democratic or authoritarian characteristics, to rate the US a non-democracy during both Trump terms [99].

Going beyond politics, some analysts argue that AGW denial helped lay the groundwork for the emergence of “alternative facts” and “post-truth,” phenomena that undermine scientific and other expertise and faith in institutions such as “mainstream media,” universities, and government agencies [100,101]. This opens the door to disinformation and conspiracies [102] such as “Covid is a hoax” or that vaccinations are dangerous widely spread by right-wing (and sometimes left-wing) media, websites and spokespersons, including the second term Trump Health and Human Services secretary, Robert F. Kennedy, Jr.

The result of these epistemological attacks is that many people suspect basic facts, making it difficult for democracy to function while endangering people with falsehoods.

Recognizing the danger of disinformation, individual researchers, universities and some NGOs have developed programs to study and develop techniques to counter disinformation, but their efforts have received a hostile reception among those who spread it —including efforts to intimidate disinformation research which the far-right labels as censorship, such as the US House Judiciary Select Subcommittee on the Weaponization of the Federal Government in the 118^th^ Congress.

These kinds of hard-core actions by key forces of anti-reflexivity have taken efforts to undermine AGW science and policies to a new level, defending disinformation of all types including those about election integrity. These escalating attacks on facts, science, and reality writ large represent “anti-reflexivity on steroids.” Thus, the efforts beginning in the 1980s of a small group of dissenting scientists, pseudo-scientists and far right journalists (including those who produced the books in our analyses), often funded by the fossil fuel industry and conservative think-tanks and promoted by ideological media, have certainly borne fruit—to the detriment of the environment and humankind.

## Supporting information

S1 Appendix ABS.D&J.Appendix.Final. (DOCX)
