# Supplementary material for: Foundations of climate change denial: Anti-environmentalism and anti-science
Source: PLoS One. 2025 Nov 12;20(11):e0334544. doi: 10.1371/journal.pone.0334544 (PMC12611160; doi:10.1371/journal.pone.0334544)
Supplement: Attachment — Submitted filename: Reviewer comments.docx [file pone.0334544.s003.docx]

We thank the editor and reviewers. Our responses to the reviewers are in bold.

Reviewer #1: The article describes an ambitious attempt to conduct a systematic qualitative analysis of a large number of books (over a hundred). This is, in itself, a convincing effort to uncover underlying themes in climate denialist literature. There arise a number of issues that need clarification. My overall assessment is between minor and major revision, and I would leave it to the editor to decide which category is more appropriate.

**We appreciate the reviewers comments and suggestions.**

One general comment is that, while the coding process done in stage one seems straightforward, it is unclear what the process exactly is in the second stage and how new themes emerged in this stage.

**We have attempted to clarify this several ways in the ms. Part of the answer is found in the coding frame “what is the denial claim” and “what is the rationale” which are stages one and two respectively. Stage two also follows co-occurrence logic—within a chapter section (we realize this detail was missing and have added it now) what co-occurs with the denial claim. So, if an author denies that polar bears are threatened by climate change because s/he 1) says the number of bears is at a historic high and 2) polar bear scientists selling scare stories are corrupt—we take #1 to be superficial and #2 to be a more fundamental rationale because if scientists are thought to be corrupt none of their answers, say about the health of bear populations, can be trusted. Normally these two messages co-occur with each other in a chapter section because that section is targeting more specific things, like polar bear futures.**

i. Corpus of Texts
What is the significance of the type of texts being analyzed (books)? I can intuit this, but perhaps this is something that needs explicit mention. Were books selected because, being treatise length texts, they are likely to contain whole narratives, completely written out, such that themes are fully developed? Are books deeper repositories of meaning, so that the "DNA" of climate denialist discourse are there to discern (but not in shorter texts such as blogs or articles)?

**Our corpus was selected by 1) rejecting one or more of the three denial claims (trend, attribution, impact) and having an ISBN. All of our corpus is also in English. This means there is a wide degree of variance in the importance of each book. Some are from elite think tank policy insiders, some are self-published. We have added a statement explaining this. We did choose books as a target for the corpus because they are longer discourses and they confer a sense of authority to the author.**

ii. First Stage
The two stage approach involves a deductive and inductive process. Isn't the deductive approach basically a thematic analysis, the a priori concepts being themes identified in previous literature? If so, perhaps the authors can clarify this.

**Yes, it is a thematic analysis. We have added this language.**

The findings from the first stage reinforce findings of previous investigations, namely four types of denial (trend, attribution, impact, policy). The discussion should answer the question: what was learned that was new or an addition to what previous works had identified? Or is the significance that these themes were almost universally found in systematic climate denial treatises (the books)?

**Indeed, we confirm layers of findings in existing literature, so stage one does not add a whole lot to the literature, but is necessary in order to identify themes in stage two (i.e., what is discussed alongside the denial claim?). But we do take from this stage that the themes were almost universally found in the books.**

Perhaps some discussion should go into sub-themes used in the thematic analysis, as these may provide a finer-grained discussion of the nature of each of the types of denial.

**Thank you, we have added language to indicate that the quotes come from sub-themes. E.g., Section 4.2 we write: “Here are representative claims made by authors in the dataset, with each bullet point indicatinga sub-theme of the larger denial category”**

The claim is made, later in the discussion of the first stage coding, that "We infer from this data that authors dismiss the role of GHGs in climate change in order to justify policy denial, because if the cause of warming is wrong, de-carbonization is unnecessary." But this higher-level, relational reasoning is something that emerges from stage two, isn't it (or perhaps I am not reading it correctly)?

**We agree this is a premature statement at stage one and have removed it.**

iii. Second Stage

On pages 15 and 16, there is a discussion of how coded text in stage one are subject to a relational type of coding in stage two. This section needs better explanation, as it is unclear how the process works. The discussion is confusing. It seems to suggest (but not at all clearly) that contiguity (of text) can be taken as logical necessity (i.e., that A ◊ B). So, text (A) that constitutes some form of denial is succeeded by text (B) about climate science being a scientific hoax. So, what exactly is the connection drawn between A and B? Unclear what is being described… is the rejected science being equated to hypocrisy on the part of the scientists? What is the narrative, exactly? And is this logical connection between A and B what the authors later refer to as the theme of anti-reflexivity found in the second stage? This part of the discussion is confusing.

**We have added the detail that the denial claim co-occurs within a chapter section and are thereby related and add meaning based on “the company they keep” (Firth 1968 citation added). We have also selected a different set of quotes from the same book (and have had to remove all the figures for copyright reasons) in figure 2 that hopefully lend better clarity to the process. We have also rewritten this section a bit to add clarity.**

**In addition, the two texts coded are contiguous within chapter sections, but are also connected semantically—authors tie the two sections together with their own language. We have also tried to clarify this more. For the anti-reflexivity finding, this comes in part from the move to stage two, but really from the combination of themes discovered in stage two. We revised to the following:**

**“Our nodes are connected by “proximities within a textual unit,” which are chapter sections in this case. We draw the connection between nodes through comments that directly and semantically tie one node to another. One node is always a statement of climate denial.” (p. 5).**

A better description is needed of how exactly the themes (or narratives) of the politicization of science and anti-environmentalism found in the second-stage coding. Are these results of the relational coding described in pages 15 and 16? It is unclear, to this reviewer, how the inductive process works. Is it akin to coding in grounded theory? It is unclear how these two underlying themes emerged.

Take the paragraph below:

"Another example shows the anti-environmental theme in Fig. 3, which shows the denial (impact) claim, the connector in “But now we come to an additional point of overriding importance” and the issue of overriding importance is the “ulterior motives” of “the environmentalists, who are against anything that has to do with industrialization and comfort provided by technological progress” (Weber 1991, emphasis added)."
It is not clear what the analysis is, exactly. What exactly is the logical connection made between A and B --can this be stated definitively? Is A taken as proof of B? Or is the claim that B is an underlying theme to A? Does most of the second stage analysis involve such connection of two texts, and is the connector taken to be almost like a set operation, so that theme A is subsumed by the deeper theme, B?

**We thank the reviewer for this overall observation because it indicates we have left some language out that should help. That language is that the authors semantically tie the two logics together themselves in their narrative within a chapter section—the denial claim and the meta-themes of anti-science and anti-environmentalism. We hope we have made this logic more transparent.**

Perhaps a more thorough/explicit explanation is needed of how, for example, the anti-environmental theme was uncovered in the second stage of the analysis. Was this one theme among the others previously found (the four types of denial), or was it a meta-theme/meta-narrative that underlies the denialist text? How did the text in stage one lead to the identification of the anti-environmentalism discourse?

**Anti-environmental is a meta-theme separate from the four CC denial claims/themes, and it was identified through recurring critiques of environmentalism in different ways (it’s a religion, it’s anti-human, anti-capitalist, etc….). The very same process identified anti-science as the other meta-theme. The consistency across the books and time was remarkable.**

The identification of anti-reflexivity (amounting to anti-environmentalism and mistrust of the scientists behind impact science) is a significant finding, but was this not already identified in previous works (such as McCright and Dunlap, 2010)? What emerged from this analysis that adds to the previous account of anti-reflexivity?

**Indeed, the reviewer is right that anti-reflexivity was identified by McCright and Dunlap in 2010. In that paper, the authors primarily analyzed anti-reflexivity in terms of non-decision making techniques and politics of the George W. Bush administration. Here, we add to this literature by covering a much longer time frame [1988? to 2010] and wider geographic representation with in-depth analysis of a large amount of data derived from a very different source—a large number of denial books--that we hope substantially adds a depth to the analysis that was not there before.**

I feel that parts of the manuscript need to be rewritten for clarity. If the textual analytic method is novel, then clarity is needed even more so.

**Thank you-- We have gone through and done an edit of the entire manuscript, looking for areas to improve clarity—including those you have raised.**

Finally, there is a missing conclusion that is maybe alluded to but not stated definitively, that what is on the surface denial of a (scientific and social) movement is, at its root, a rejection of the alien other (whether labelled anti-capitalist, socialist, etc.). Which means that the different types of denial are contrived, masking a deeper agenda.

**We have re-written the conclusion, hopefully answering this point. We agreed there needed to be a better summary. Here is part of it:**

**Anti-reflexivity is a powerful defensive mechanism employed by dominant interests to protect power and privilege ensconced in global capitalism fueled by cheap hydrocarbons and is consistent with far right nationalism, the protection of “petro-masculinity,” and white power. Dagget writes: “For many, extracting and burning fuel was a practice of white masculinity, and of American sovereignty, such that the explosive power of combustion could be crudely equated with virility” [15]. Likewise, Anshelm and Hultman [89] report, “These climate sceptics tried to save an industrial society of which they were a part by defending its values against ecomodern hegemony” (p. 84). Allowing meaningful restrictions to fossil fuels endangers the power of petro-privilege.**

**Hultman et. al. agree: “…far-right nationalism and AGW denialism [are] based upon the ideological similarities in viewing the world from an industrial masculinities viewpoint, not wanting to let go of the colonial extractive logic that has served these men well, but violated the planet” [90]. As Malm and the Zetkin Collective [14] show, following Griffin [91], far-right ideology, including fascism, has a historical relation with denial, lies, and misinformation since Mussolini. This helps us theoretically ground misinformation (not intended to deceive) and disinformation (intended to deceive)	as forces that are capable of redistributing power and obscuring alternatives, “as when a traveler is falsely told a bridge collapsed” [92] notes Bok, and in this case our bridge is limiting fossil fuels to preserve a habitable planet.**

**We thank the reviewer for their considerate effort to help us improve this paper.**

Reviewer #2: Thank you for the opportunity to review this very interesting article. The topic is highly significant --the article addresses the compelling issue of denial within the rapidly expanding field of study of climate obstruction.
Yet, the manuscript would benefit from further clarifications, particularly in the use of some key terms, while its engagement with parallel streams of literature will help strengthening the clarity and robustness of the main argument.

Title
Section 5.2 on Anti-environmentalism is crucial. Indeed, it could become part of the title itself as the theme of the far-right’s anti-environmentalism is much more central. Perhaps change the main title from ‘Defending the Western way of life’ (a rather underdeveloped argument within the article) into ‘The new far-right anti-environmentalism’ or, more bluntly, ‘Fighting against the environment’. I think this would much more concisely render the core of the article's argument and make improve its interdisciplinary accessibility.
Alternatively, if the authors choose to keep ‘Defending the Western way of life’ as the main title, then it may be worth going deeper into the colonial and imperial angles of notions such as Western or Westernisation, in which case an additional set of literature would be needed.

**We thank the reviewer here for observing that the title needs some repurposing. We are now using the title: “Foundations of Climate Change Denial: Anti-Environmentalism and Anti-Science”**

General comments
Is the term "conservative" correct when analyzing a political movement permeated by an extreme form of nationalism? Would another concept be more suitable to better convey the article's cogent message? More specifically, wouldn't far-right politics be a preferable term? This ignores the changes over decades in the CCCM. I don’t think that ‘far-right’ forces led the CCCM in the first two decades. The CoC and trade associations, e.g., were not part of a ‘far-right’ movement. But the CCCM was opposed by F

**We appreciate this overall point, nationalism is clearly in our data but we did not frame it that way. We have gone through the paper and worked to see where “far-right” and nationalism can properly be added.**

**Here is part of our treatment:**

**The CCCM was launched by fossil fuel corporations and other corporate actors dependent on fossil fuel use to protect their interests and was quickly joined by the US Conservative Movement well established in the literature (Beder, 1999; A. McCright & Dunlap, 2000; Union for Concerned Scientists, 2015). This effort has evolved over time from mainstream conservative efforts (e.g., protecting markets from regulation) to now include more far-right programs. In the US this started with the Tea Party movement (Skocpol, 2014). The literature indicates that the climate denial connection to the far-right include white power (whether stated or implied) (Blee & Yates, 2015; Lichtman, 2008), authoritarian tendencies (Bryant & Farrell, 2024) as well as specific far-right alliances to the fossil fuel industry (Krange, Kaltenborn, & Hultman, 2019) of contemporary climate denial (Daggett, 2018; Malm & Collective, 2021).**

**Ekberg writes that climate denial has “clear signs of alliance with far-right actors”(Ekberg, Forchtner, Hultman, & Jylhä, 2022). Literature characterizing the “far-right” (Pirro, 2023), argues that the label “conservative” underappreciates the AGW denial is part of the CCCM’s reactionary effort to protect the status quo against progressive interests calling for change, especially in American society and protect the socioeconomic system based on a free-market economy with its promise of endless growth and unlimited progress-- to defend the “Western way of life” writ large (Jacques, 2006; Jacques, Dunlap, & Freeman, 2008; Klein, 2015). It is increasingly recognized that the roots of AGW denial thus go much deeper than protecting the fossil fuel industry, which has literally fueled economic growth in the U.S. and much of the rest of the world for well over a century (Dunlap & Brulle, 2020; Raul P. Lejano & Dodge, 2017; Raul P Lejano & Nero, 2020). Yet, analyses of the claims used by the CCCM have not yet sufficiently verified these speculations.**

I note (and, I should add, find it hardly comprehensible) that the very notion of far-right is not mentioned once. Nor can I see any mention of the broader political right as such, except on line 743 when it appears in the context of the "Covid hoax" spread by right-wing (together with left-wing?) media.

Throughout the text, the authors prefer to use the notion of "conservative movement”, yet a crucial question arises: is the term ‘conservative’ appropriate here? It is worth asking: “who conserves what?”. From Edmund Burke and Joseph de Maistre on, 'conservative’ refers to conserving the privileges of an elite and a few values associated with it, generally presented as ‘tradition’.
**Indeed we agree that the notions of the far right nationalism needed to be added as above, and we appreciate the additional references provided in the reviewers comments—we have consulted them and added them where it seemed to make sense, along with an added discussion of the far right and nationalism. We see this as an evolutionary change where the far-right has been added to the Conservative Movement identified in much of the literature on the CCCM (e.g., Brulle, 2019; A. McCright & Dunlap, 2003; A. M. McCright & Dunlap, 2012).**

In other words, the term ‘conservative’ was the very term chosen by ‘conservatives’ themselves. Therefore, far-right politics(more specifically than right-wing politics, despite the strong association between the two) would be more appropriate, not least because of fascism’s historical association with denial, lies, and misinformation since its foundation by Benito Mussolini (see 'Eco-fascism: an oxymoron? Far-right nationalism, history, and the climate emergency', Frontiers in Human Dynamics, 6, 2024)
**Here we have added:**

**As Malm and the Zetkin Collective (Malm & Collective, 2021) show, following Griffin (Griffin, 2006), far-right ideology, including fascism, has a historical relation with denial, lies, and misinformation since Mussolini. This helps us theoretically ground misinformation (not intended to deceive) and disinformation (intended to deceive)	as forces that are capable of redistributing power and obscuring alternatives, “as when a traveler is falsely told a bridge collapsed” (Bok, 2011, p. 19) notes Bok, and in this case our bridge is limiting fossil fuels to preserve a habitable planet.**

**Also, far-right ideology is palingenic, that is, obsessed with re-birth, and AGW denial can be a re-birth of that privilege that comes through the data with nostalgia for a time when white patriarchy was fueled with abundant fossil fuels. Under this lens, greatness and progress need to be defended and reclaimed from environmentalists and scientists. Anti-reflexivity provides a rationale to reclaim this “stolen pride” —something supporters of US President Donald Trump directly articulated to sociologist Arlie Russell Hochschild, speaking from the poorest county in the US while the president’s 2025 budget removed social safety-net essentials to pay for tax credits for the wealthy (Hochschild, 2025).**

**We have also added this language and appreciate the reviewer for pointing this specific element out. We wrote at the end of Section 4.5 : “Note that some of these fears, like a fear that mitigation will arrest Western civilization progress, reproduces fascist ideology and far-right nationalist fears found in the Belle Époque (late 1900s) by the ultranationalist futurist movement (Conversi, 2009, 2024).**”

One common denominator of both contemporary far-right politics and historical fascism is the pervasiveness of nationalism, sometimes identified as ultra-nationalism, as works by Andreas Malm, following Roger Griffin (see White skin, black fuel: On the danger of fossil fascism. London: Verso, 2021).

**Thank you, we have added both of these references with discussion.**

I note that nationalism is never mentioned in the article, and, I think, this is the key missing link needed to assemble the pieces of the puzzle together. Indeed, until very recently, nationalism scholars themselves have been nearly oblivious to climate change, a meaningful absence that needs to be addressed. The first scholarly article to address the deep linkage between the two has only appeared in 2020: 'The ultimate challenge: Nationalism and climate change', Nationalities Papers, 2020

**We have added a discussion on nationalism in the conclusion now (the quote above on page 32)**

- Comments and a few critical remarks
  The correct observation that neoliberal and far-right's claim that "environmentalism is akin to totalitarian fascism" (642) is worth expanding. Yet, similar claims are sometime shared by circles and currents within the far-left, even among a few scholars, particularly political geographers (once more, see the first article mentioned above for a historical perspective).
  **We have added a sentence along with the Conversi 2020 source below on resource nationalism.** **“Environmentalism is akin to totalitarian fascism. This probably comes from resource nationalism, where hydrocarbons are fetishized and limits to using them becomes a form of excising national identity (Conversi, 2020).” P. 26r**
- As for reflexivity and anti-reflexivity (136-ff. 286-ff), this is fine-tuned with Dunlap’s 2014 Environmental Research Letters brief Perspective piece and finely expands on it. But how can it help understanding the complexity of the current political landscape? In this respect, and connecting again with the central nationalist component, the concept of “reflexive green nationalism" may perhaps be usefully incorporated. The reflexivity of "green nationalism" has been considered as a wholly alternative option vis a vis far-right’s anti-reflexivity.
  **We have attempted to put a finer point on the current political landscape with more discussion about misinformation generally and discussion of the Trump administration:**

**“Yet, the Trump Administration in less than two terms has exceeded all prior efforts to deny the reality and dangers of climate change by openly and aggressively attacking climate change policies, withdrawing the US from the Paris Agreement, and sabotaging international cooperation to reduce carbon emissions, putting climate change deniers like Scott Pruitt in charge of agencies such as the Environmental Protection agency in the first term, promoting energy production with no regard to environmental consequences, and much more (Turner & Isenberg, 2018). And crucially, Trump normalized labeling climate change a “hoax” and a “scam” among Republican politicians, reflecting the success of the CCCM’s efforts to instill hardcore denial into one of our nation’s two major political parties (or at least what remains of the GOP under Trump). In July of 2025 during his second term, the Trump administration hired two authors in our data, John Christy and Roy Spencer—and one author who likely would be in our data if it extended to 2021, Steven E. Koonin, to the Energy Department. All of this speaks to an agenda to defend the structures of Western, white, nativist power from a global problem many believe cannot be solved if that very system remains in place (DeLay, 2024; Petersen, Stuart, & Gunderson, 2019). Indeed, these far-right moves have led the Polity project, which rates countries for democratic or authoritarian characteristics, to rate the US a non-democracy during both Trump terms (Goldenberg, 2025).”**

The appropriate notion of pseudo-scientist appears more than once, but then why using the term “dissident scientists” (760)? The idea of free dissident is a badge of honour conferred upon those who spread misinformation, rather than rationally dissenting from science.
**We have changed this language to “dissenting”**

The term ‘misinformation’ only appears three times (191, 747, 754), far less than one should expect in an article devoted to analyse a movement based on the spread of misinformation. The term ‘disinformation’ also appears three times (741, 745, 750), but this is clearly dissectible form ‘misinformation’ (see ‘From climate change denial to war-mongering nationalism’, Cultures of Sustainability/ Culture della Sostenibilità, 31: 99-122.2023)
**We have added (after discussing Kyoto):**

**These disinformation tactics only became increasingly successful when the Conference of Parties met in Copenhagen with the hope that the Kyoto Protocol could replaced with an equally serious institution. The called “Hopenhagen” meeting was a,**

**…pivotal moment in history when nationalist and corporate forces formed new alliances and sought novel ways to undermine climate resolutions. The denial machine’s ability to hinder cooperative and coordinated efforts between nation-states was demonstrated through an unprecedented attack on the summit using media and social media platforms to disseminate disinformation and fuel denialism(CONVERSI, HASSAN, & POSOCCO, 2023, p. abstract).**

I find the far-right’s new "bête noire" of environmentalism as “the new socialist enemy to freedom’ (629) most relevant, central and inspiring. But it is well worth expanding on various dimensions of far-right politics, particularly utra-nationalism and neoliberalism — ’Capitalism and nationalism’, Ethnic and Racial Studies, 46 (8), 2023: 1685-1690. While not all nationalists are anti-environmentalists, and it may thus be possible to speak of "green nationalism" (see the recent booming literature on this), it is worth considering in depth the various linkages existing between far-right politics and anti-environmentalism.

The very notion of ‘movement’ applied to the CCCM also warrants closer scrutiny: depending on the emphasis and definition, the term ‘social movement’ may imply a spontaneous grassroots component. But this is an effort that may be worth pursuing in another article.

**We take the reviewers point, and see the potential confusion-- the CCCM is a counter-movement, organized by elites to combat a social movement that threatens to successfully create change. To clarify, we add:**

**“A social movement is a conscious collective effort to organize for social change. If a movement threatens to be successful, elites often will organize against the social movement in a “countermovement”(Lo, 1982; Meyer & Staggenborg, 1996). As the global environmental movement threatened to successfully convince the international community to fight climate change and thereby threaten the political-economic status quo, elites organized an opposition project, a counter-movement against the environmental movement. This is the climate change countermovement (CCCM).” P. 6**

Minor comments
Line 30: “skeptical scientists that has successfully thwarted …”. The term “skeptical” has been criticized as it seems to grant an aura of respectability and even a scientific pedigree on those who disseminate antiscientific information. Yet, on lines 34 -37 you recognise their effort in “rejecting the basic findings of climate science” and that they “cast the integrity of climate science and scientists in doubt”. The term “anti-science” would be clearer.

**We have replaced “skeptical” throughout the ms with denier or dissenter except where it appears in a quote.**

35-36 The distinction between (trend denial), (attribution denial), (impact denial), and (policy denial) is very useful and essential.

51 -52 on the 97% consensus among climate scientists about human-caused greenhouse gas (GHGs) emissions, the percentage is apparently 100% - see ‘Scientists Reach 100% Consensus on Anthropogenic Global Warming, Bulletin of Science, Technology & Society, 2017, 37 (4): 183-184’.

**We emphasize the “more than 99%” finding now.**

60 when you rightly say that the CCCM was launched by fossil fuel corporations some references would perhaps be useful, while indications of the composition of the US Conservative Movement would also help. For instance, how far was the Tea party central here? See . The Tea Party and the Remaking of Republican Conservatism, Perspectives on Politics, 9 (1) 2011: 25-43 and. The Koch Network and Republican Party Extremism, Perspectives on Politics, 14 (3): 681-699, 2016.

Less important is ’Denial, deflection, and distraction: Neutralizing charges of racism by the Tea Party movement’, Mobilization, more superficial, but here the central notion of ‘distraction’ is briefly presented. In ongoing research, I have noted how ‘distraction’ can provide a broader and perhaps more explicative concept that climate obstruction itself.

67 Yes, it may be that “the roots of AGW denial thus go much deeper than protecting the fossil fuel industry”, but when did the movement begin? Wasn’t the fossil fuels industry that launched it? Why not using the notion of “fossil fuel complex”, which would include many other industrial actors.
**We have added context after the explanation of what a social and countermovement is, “This is the CCCM and it started in 1992 after the Rio “Earth” Summit announced global environmental problems and the collapse of the Soviet Union left anti-communist activists without an agenda (Jacques et al., 2008).”** **P. 6.**

Final recommendations
The underlying question is whether another set of data, however impeccable, may add substantially new information to the field. The field itself still lacks a broader historically grounded theoretical perspective about denial, disinformation and misinformation.
If the concepts used are inadequate or poorly explained, then the entire datasets associated with them risk loosing their scientific value, particularly if associated with interdisciplinarity, transdisciplinarity and cross-disciplinarity. That's why a conceptual deepening and a face-to-face assessment of the existing terminology should be a sine qua non requirement for publication. As the article is sufficiently robust and informative, this could be done quite easily without major revisions.
**We have added comments about a theoretical perspective for disinformation around the discussion of privilege defending:**

**“Allowing meaningful restrictions to fossil fuels endangers the power of petro-privilege. This helps us theoretically ground misinformation (not intended to deceive) and disinformation (intended to deceive) as a force that has the capability to redistribute power and obscure alternatives, “as when a traveler is falsely told a bridge collapsed” (Bok, 2011, p. 19) notes Bok, and in this case our bridge is limiting fossil fuels** **to preserve a habitable planet.” Pp. 30-31.**

In short, this article is highly worth publishing and will doubtlessly have a high impact across disciplines. Such impact can be increased with some conceptual clarifications and amendments, particularly in two areas: first, the addition of two/three paragraphs highlighting the importance of nationalism and, secondly, attentively considering how and why the term ‘far-right’ is more appropriate than ‘conservative movement’ — at least in the sense in which both terms are intended beyond the USA.

**We appreciate the attention the reviewer has given to our article. We have added throughout the paper attention to nationalism and the far-right while we removed “conservative movement” except where it is in a quote and where we try to put more context in the discussion.**

**We write in the introduction:**

**The CCCM was launched by fossil fuel corporations and other corporate actors dependent on fossil fuel use to protect their interests and was quickly joined by the US Conservative Movement well established in the literature (Beder, 1999; A. McCright & Dunlap, 2000; Union for Concerned Scientists, 2015).** **This effort has evolved over time from mainstream conservative efforts (e.g., protecting markets from regulation) to now include more far-right programs. In the US this started with the Tea Party movement (Skocpol, 2014). The literature indicates that the climate denial connection to the far-right include white power (whether stated or implied) (Blee & Yates, 2015; Lichtman, 2008), authoritarian tendencies (Bryant & Farrell, 2024) as well as specific far-right alliances to the fossil fuel industry (Krange et al., 2019) of contemporary climate denial (Daggett, 2018; Malm & Collective, 2021).**

**Ekberg writes that climate denial has “clear signs of alliance with far-right actors”(Ekberg et al., 2022). Literature characterizing the “far-right” (Pirro, 2023), argues that the label “conservative” underappreciates the AGW denial is part of the CCCM’s reactionary effort to protect the status quo against progressive interests calling for change, especially in American society and protect the socioeconomic system based on a free-market economy with its promise of endless growth and unlimited progress-- to defend the “Western way of life” writ large (Jacques, 2006; Jacques et al., 2008; Klein, 2015). It is increasingly recognized that the roots of AGW denial thus go much deeper than protecting the fossil fuel industry, which has literally fueled economic growth in the U.S. and much of the rest of the world for well over a century (Dunlap & Brulle, 2020; Raul P. Lejano & Dodge, 2017; Raul P Lejano & Nero, 2020). Yet, analyses of the claims used by the CCCM have not yet sufficiently verified these speculations.**

Reviewer #3: I am a physical climate scientist rather than a social scientist, but I am extremely familiar with the climate denial environment. Overall, I don't see any problems with this paper. All of the results seem sound, and I have to say that what they accomplished required an enormous amount of work.

My only concern with the paper is that the books they analyzed have publication dates no later than 2010. Based on my own anecdotal experience, explicit climate denial—such as trend denial or attribution denial—has largely faded away since then. Now, prominent deniers such as Alex Epstein basically make arguments about what's best for humanity or Steve Koonin, who make value-based arguments ("The science is too unsettled for us to act") that sound like scientific arguments but are not, or Bjorn Lomborg, who makes arguments about the economics of climate change. None of these are really scientific arguments, although these people do their best to make them sound scientific.

My guess is that if you had a sufficiently long data set ending closer to today and conducted a time series analysis, you would see an interesting evolution of denial.

I don't have any specific suggestions for the authors, as I don't think it would be reasonable for me to demand they extend their analysis to include books published later. However, it would IMHO be useful to pursue that in future work.

**We thank the reviewer for these thoughtful comments. We also wish the data went to a later year, and we do hope to extend the analysis at some point to look for the patterns mentioned here.**

Beder, S. (1999). Climatic confusion and corporate collusion: hijacking the greenhouse debate. *The Ecologist, 1999*, 119-122.

Blee, K. M., & Yates, E. A. (2015). The Place of Race in Conservative and Far-right Movements. *Sociology of Race and Ethnicity, 1*(1), 127-136. doi:10.1177/2332649214555031

Bok, S. (2011). *Lying: Moral choice in public and private life*: Vintage.

Brulle, R. J. (2019). Networks of Opposition: A Structural Analysis of U.S. Climate Change Countermovement Coalitions 1989–2015. *Sociological Inquiry, n/a*(n/a). doi:10.1111/soin.12333

Bryant, J. C., & Farrell, J. (2024). Conservatism, the far right, and the environment. *Annual Review of Sociology, 50*(1), 273-296.

Conversi, D. (2009). Art, nationalism and war: Political futurism in Italy (1909–1944). *Sociology Compass, 3*(1), 92-117.

Conversi, D. (2020). The Ultimate Challenge: Nationalism and Climate Change. *Nationalities Papers, 48*(4), 625-636. doi:10.1017/nps.2020.18

Conversi, D. (2024). Eco-fascism: an oxymoron? Far-right nationalism, history, and the climate emergency. *Frontiers in Human Dynamics, 6*, 1373872.

CONVERSI, D., HASSAN, C., & POSOCCO, L. (2023). From climate change denial to war-mongering nationalism. *International journal of political ecology, 31*, 99-122.

Daggett, C. (2018). Petro-masculinity: Fossil fuels and authoritarian desire. *Millennium, 47*(1), 25-44.

DeLay, T. (2024). *Future of Denial: The Ideologies of Climate Change*: Verso Books.

Dunlap, R. E., & Brulle, R. J. (2020). Sources and amplifiers of climate change denial. In *Research Handbook on Communicating Climate Change*: Edward Elgar Publishing.

Ekberg, K., Forchtner, B., Hultman, M., & Jylhä, K. M. (2022). *Climate obstruction: How denial, delay and inaction are heating the planet*: Routledge.

Goldenberg, G. (2025). From Crisis to Action: Turning the Tide on Democratic Erosion Through Organizing. Retrieved from <https://ash.harvard.edu/articles/from-crisis-to-action-turning-the-tide-on-democratic-erosion-through-organizing/>

Griffin, R. (2006). *The nature of fascism*. New York: Routledge.

Hochschild, A. (2025). My Journey Deep in the Heart of Trump Country. *The New York Times*. Retrieved from <https://www.nytimes.com/2025/06/09/opinion/trump-supporters-kentucky.html>

Jacques, P. J. (2006). The Rearguard of Modernity: Environmental Skepticism as a Struggle of Citizenship. *Global Environmental Politics, 6*(1), 76-101. doi:10.1162/glep.2006.6.1.76

Jacques, P. J., Dunlap, R. E., & Freeman, M. (2008). The Organization of Denial: Conservative Think Tanks and Environmental Scepticism. *Environmental Politics, 17*(3), 349 — 385. doi:10.1080/09644010802055576

Klein, N. (2015). *This changes everything: Capitalism vs. the climate*: Simon and Schuster.

Krange, O., Kaltenborn, B. P., & Hultman, M. (2019). Cool dudes in Norway: climate change denial among conservative Norwegian men. *Environmental Sociology, 5*(1), 1-11.

Lejano, R. P., & Dodge, J. (2017). The narrative properties of ideology: the adversarial turn and climate skepticism in the USA. *Policy Sciences, 50*(2), 195-215. doi:10.1007/s11077-016-9274-9

Lejano, R. P., & Nero, S. J. (2020). *The Power of Narrative: Climate Skepticism and the Deconstruction of Science*: Oxford University Press, USA.

Lichtman, A. J. (2008). *White Protestant nation: The rise of the American conservative movement*: Grove Press.

Lo, C. Y. H. (1982). Countermovements and Conservative Movements in the Contemporary U.S. *Annual Review of Sociology, 8*, 107-134.

Malm, A., & Collective, Z. (2021). *White skin, black fuel: On the danger of fossil fascism*: Verso Books.

McCright, A., & Dunlap, R. E. (2000). Challenging Global Warming as a Social Problem: An Analysis of the Conservative Movement’s Counter Claims. *Social Problems, 47*(4), 499–522.

McCright, A., & Dunlap, R. E. (2003). Defeating Kyoto: The Conservative Movement’s Impact on U.S. Climate Change Policy. *Social Problems, 50*(3), 348–373.

McCright, A. M., & Dunlap, R. E. (2012). Bringing ideology in: the conservative white male effect on worry about environmental problems in the USA. *Journal of Risk Research, 16*(2), 211-226. doi:10.1080/13669877.2012.726242

Meyer, D. S., & Staggenborg, S. (1996). Movements, Countermovements, and the Structure of Political Opportunity. *The American Journal of Sociology, 101*(6), 1628-1660.

Petersen, B., Stuart, D., & Gunderson, R. (2019). Reconceptualizing climate change denial. *Human Ecology Review, 25*(2), 117-142.

Pirro, A. L. (2023). Far right: The significance of an umbrella concept. *Nations and Nationalism, 29*(1), 101-112.

Skocpol, T. (2014). Re: Making sense of the past and future politics of global warming in the United States

Turner, J. M., & Isenberg, A. C. (2018). *The Republican reversal: conservatives and the environment from Nixon to Trump*: Harvard University Press.

Union for Concerned Scientists. (2015). *The Climate Deception Dossiers: Internal Fossil Fuel Industry Memos Reveal Decades of Corporate Disinformation*. Retrieved from Washington, D.C.: <https://www.ucsusa.org/resources/climate-deception-dossiers>
