# Supplementary material for: Foundations of climate change denial: Anti-environmentalism and anti-science
Source: PLoS One. 2025 Nov 12;20(11):e0334544. doi: 10.1371/journal.pone.0334544 (PMC12611160; doi:10.1371/journal.pone.0334544)
Supplement: Peer review history

# Peer review: Decision Letter 0

**DOI:** 10.1371/journal.pone.0334544.r001

**Authors:** Rahul Sambaraju

19 Jun 2025

PONE-D-24-48750Defending the Western way of life: Uncovering the fundamental rationale for climate change denialPLOS ONE

Dear Dr. Jacques,

Thank you for submitting your manuscript to PLOS ONE. After careful consideration, we feel that it has merit but does not fully meet PLOS ONE’s publication criteria as it currently stands. Therefore, we invite you to submit a revised version of the manuscript that addresses the points raised during the review process.

Please submit your revised manuscript by Aug 03 2025 11:59PM. If you will need more time than this to complete your revisions, please reply to this message or contact the journal office at plosone@plos.org . When you're ready to submit your revision, log on to https://www.editorialmanager.com/pone/ and select the 'Submissions Needing Revision' folder to locate your manuscript file.

Please include the following items when submitting your revised manuscript:

- A rebuttal letter that responds to each point raised by the academic editor and reviewer(s). You should upload this letter as a separate file labeled 'Response to Reviewers'.
- A marked-up copy of your manuscript that highlights changes made to the original version. You should upload this as a separate file labeled 'Revised Manuscript with Track Changes'.
- An unmarked version of your revised paper without tracked changes. You should upload this as a separate file labeled 'Manuscript'.

If you would like to make changes to your financial disclosure, please include your updated statement in your cover letter. Guidelines for resubmitting your figure files are available below the reviewer comments at the end of this letter.

If applicable, we recommend that you deposit your laboratory protocols in protocols.io to enhance the reproducibility of your results. Protocols.io assigns your protocol its own identifier (DOI) so that it can be cited independently in the future. For instructions see: https://journals.plos.org/plosone/s/submission-guidelines#loc-laboratory-protocols . Additionally, PLOS ONE offers an option for publishing peer-reviewed Lab Protocol articles, which describe protocols hosted on protocols.io. Read more information on sharing protocols at https://plos.org/protocols?utm_medium=editorial-email&utm_source=authorletters&utm_campaign=protocols .

We look forward to receiving your revised manuscript.

Kind regards,

Rahul Sambaraju

Academic Editor

PLOS ONE

Journal Requirements:

When submitting your revision, we need you to address these additional requirements.

1. Please ensure that your manuscript meets PLOS ONE's style requirements, including those for file naming. The PLOS ONE style templates can be found at

https://journals.plos.org/plosone/s/file?id=wjVg/PLOSOne_formatting_sample_main_body.pdf and

https://journals.plos.org/plosone/s/file?id=ba62/PLOSOne_formatting_sample_title_authors_affiliations.pdf

2. If published, this research might receive public and media attention; such conclusions may also be subject to over-interpretation or misunderstanding. If you are considering this work for eventual publication, perhaps following revision, we would strongly encourage you to ensure it is evaluated by at least two external reviewers, and that you take care to be sure all statements made are thoroughly supported by the results. If you would like assistance from a statistical reviewer (one of http://journals.plos.org/plosone/s/advisory-groups#loc-statistical-advisors), please email plosone@plos.org to request this. If you feel the manuscript should be rejected without review, please clearly state your reasons for the decision in your letter to the authors, as is the requirement for all rejections. If you would like to discuss this work, or if you would like suggestions of peer reviewers, please email plosone@plos.org .

3. We note that Figure(s) 1, 2, and 3 in your submission contain copyrighted images. All PLOS content is published under the Creative Commons Attribution License (CC BY 4.0), which means that the manuscript, images, and Supporting Information files will be freely available online, and any third party is permitted to access, download, copy, distribute, and use these materials in any way, even commercially, with proper attribution. For more information, see our copyright guidelines: http://journals.plos.org/plosone/s/licenses-and-copyright.

We require you to either (a) present written permission from the copyright holder to publish these figures specifically under the CC BY 4.0 license, or (b) remove the figures from your submission:

a. You may seek permission from the original copyright holder of Figure(s) 1, 2, and 3 to publish the content specifically under the CC BY 4.0 license.

We recommend that you contact the original copyright holder with the Content Permission Form (http://journals.plos.org/plosone/s/file?id=7c09/content-permission-form.pdf) and the following text:

“I request permission for the open-access journal PLOS ONE to publish XXX under the Creative Commons Attribution License (CCAL) CC BY 4.0 (http://creativecommons.org/licenses/by/4.0/). Please be aware that this license allows unrestricted use and distribution, even commercially, by third parties. Please reply and provide explicit written permission to publish XXX under a CC BY license and complete the attached form.”

Please upload the completed Content Permission Form or other proof of granted permissions as an ""Other"" file with your submission.

In the figure caption of the copyrighted figure, please include the following text: “Reprinted from [ref] under a CC BY license, with permission from [name of publisher], original copyright [original copyright year].”

b. If you are unable to obtain permission from the original copyright holder to publish these figures under the CC BY 4.0 license or if the copyright holder’s requirements are incompatible with the CC BY 4.0 license, please either i) remove the figure or ii) supply a replacement figure that complies with the CC BY 4.0 license. Please check copyright information on all replacement figures and update the figure caption with source information. If applicable, please specify in the figure caption text when a figure is similar but not identical to the original image and is therefore for illustrative purposes only.

4. Please include captions for your Supporting Information files at the end of your manuscript, and update any in-text citations to match accordingly. Please see our Supporting Information guidelines for more information: http://journals.plos.org/plosone/s/supporting-information.

5. Please remove all personal information, ensure that the data shared are in accordance with participant consent, and re-upload a fully anonymized data set.

Note: spreadsheet columns with personal information must be removed and not hidden as all hidden columns will appear in the published file.

Additional guidance on preparing raw data for publication can be found in our Data Policy (https://journals.plos.org/plosone/s/data-availability#loc-human-research-participant-data-and-other-sensitive-data) and in the following article: http://www.bmj.com/content/340/bmj.c181.long.

6. Please review your reference list to ensure that it is complete and correct. If you have cited papers that have been retracted, please include the rationale for doing so in the manuscript text, or remove these references and replace them with relevant current references. Any changes to the reference list should be mentioned in the rebuttal letter that accompanies your revised manuscript. If you need to cite a retracted article, indicate the article’s retracted status in the References list and also include a citation and full reference for the retraction notice.

[Note: HTML markup is below. Please do not edit.]

Reviewers' comments:

Reviewer's Responses to Questions

**Comments to the Author**

1. Is the manuscript technically sound, and do the data support the conclusions?

The manuscript must describe a technically sound piece of scientific research with data that supports the conclusions. Experiments must have been conducted rigorously, with appropriate controls, replication, and sample sizes. The conclusions must be drawn appropriately based on the data presented. 

Reviewer #1: Partly

Reviewer #2: Yes

Reviewer #3: Yes

**********

2. Has the statistical analysis been performed appropriately and rigorously? 

Reviewer #1: N/A

Reviewer #2: Yes

Reviewer #3: N/A

**********

3. Have the authors made all data underlying the findings in their manuscript fully available?

The PLOS Data policy requires authors to make all data underlying the findings described in their manuscript fully available without restriction, with rare exception (please refer to the Data Availability Statement in the manuscript PDF file). The data should be provided as part of the manuscript or its supporting information, or deposited to a public repository. For example, in addition to summary statistics, the data points behind means, medians and variance measures should be available. If there are restrictions on publicly sharing data—e.g. participant privacy or use of data from a third party—those must be specified.

Reviewer #1: No

Reviewer #2: Yes

Reviewer #3: Yes

**********

4. Is the manuscript presented in an intelligible fashion and written in standard English?

PLOS ONE does not copyedit accepted manuscripts, so the language in submitted articles must be clear, correct, and unambiguous. Any typographical or grammatical errors should be corrected at revision, so please note any specific errors here.

Reviewer #1: No

Reviewer #2: Yes

Reviewer #3: Yes

**********

5. Review Comments to the Author

Please use the space provided to explain your answers to the questions above. You may also include additional comments for the author, including concerns about dual publication, research ethics, or publication ethics. (Please upload your review as an attachment if it exceeds 20,000 characters)

Reviewer #1: The article describes an ambitious attempt to conduct a systematic qualitative analysis of a large number of books (over a hundred). This is, in itself, a convincing effort to uncover underlying themes in climate denialist literature. There arise a number of issues that need clarification. My overall assessment is between minor and major revision, and I would leave it to the editor to decide which category is more appropriate.

One general comment is that, while the coding process done in stage one seems straightforward, it is unclear what the process exactly is in the second stage and how new themes emerged in this stage.

i. Corpus of Texts

What is the significance of the type of texts being analyzed (books)? I can intuit this, but perhaps this is something that needs explicit mention. Were books selected because, being treatise length texts, they are likely to contain whole narratives, completely written out, such that themes are fully developed? Are books deeper repositories of meaning, so that the "DNA" of climate denialist discourse are there to discern (but not in shorter texts such as blogs or articles)?

ii. First Stage

The two stage approach involves a deductive and inductive process. Isn't the deductive approach basically a thematic analysis, the a priori concepts being themes identified in previous literature? If so, perhaps the authors can clarify this.

The findings from the first stage reinforce findings of previous investigations, namely four types of denial (trend, attribution, impact, policy). The discussion should answer the question: what was learned that was new or an addition to what previous works had identified? Or is the significance that these themes were almost universally found in systematic climate denial treatises (the books)?

Perhaps some discussion should go into sub-themes used in the thematic analysis, as these may provide a finer-grained discussion of the nature of each of the types of denial.

The claim is made, later in the discussion of the first stage coding, that "We infer from this data that authors dismiss the role of GHGs in climate change in order to justify policy denial, because if the cause of warming is wrong, de-carbonization is unnecessary." But this higher-level, relational reasoning is something that emerges from stage two, isn't it (or perhaps I am not reading it correctly)?

iii. Second Stage

On pages 15 and 16, there is a discussion of how coded text in stage one are subject to a relational type of coding in stage two. This section needs better explanation, as it is unclear how the process works. The discussion is confusing. It seems to suggest (but not at all clearly) that contiguity (of text) can be taken as logical necessity (i.e., that A � B). So, text (A) that constitutes some form of denial is succeeded by text (B) about climate science being a scientific hoax. So, what exactly is the connection drawn between A and B? Unclear what is being described… is the rejected science being equated to hypocrisy on the part of the scientists? What is the narrative, exactly? And is this logical connection between A and B what the authors later refer to as the theme of anti-reflexivity found in the second stage? This part of the discussion is confusing.

A better description is needed of how exactly the themes (or narratives) of the politicization of science and anti-environmentalism found in the second-stage coding. Are these results of the relational coding described in pages 15 and 16? It is unclear, to this reviewer, how the inductive process works. Is it akin to coding in grounded theory? It is unclear how these two underlying themes emerged.

Take the paragraph below:

"Another example shows the anti-environmental theme in Fig. 3, which shows the denial (impact) claim, the connector in “But now we come to an additional point of overriding importance” and the issue of overriding importance is the “ulterior motives” of “the environmentalists, who are against anything that has to do with industrialization and comfort provided by technological progress” (Weber 1991, emphasis added)."

It is not clear what the analysis is, exactly. What exactly is the logical connection made between A and B --can this be stated definitively? Is A taken as proof of B? Or is the claim that B is an underlying theme to A? Does most of the second stage analysis involve such connection of two texts, and is the connector taken to be almost like a set operation, so that theme A is subsumed by the deeper theme, B?

Perhaps a more thorough/explicit explanation is needed of how, for example, the anti-environmental theme was uncovered in the second stage of the analysis. Was this one theme among the others previously found (the four types of denial), or was it a meta-theme/meta-narrative that underlies the denialist text? How did the text in stage one lead to the identification of the anti-environmentalism discourse?

The identification of anti-reflexivity (amounting to anti-environmentalism and mistrust of the scientists behind impact science) is a significant finding, but was this not already identified in previous works (such as McCright and Dunlap, 2010)? What emerged from this analysis that adds to the previous account of anti-reflexivity?

I feel that parts of the manuscript need to be rewritten for clarity. If the textual analytic method is novel, then clarity is needed even more so.

Finally, there is a missing conclusion that is maybe alluded to but not stated definitively, that what is on the surface denial of a (scientific and social) movement is, at its root, a rejection of the alien other (whether labelled anti-capitalist, socialist, etc.). Which means that the different types of denial are contrived, masking a deeper agenda.

Works Cited

McCright, A. M., & Dunlap, R. E. (2010). Anti-reflexivity. Theory, Culture & Society, 27(2-3), 100-133.

Reviewer #2: Thank you for the opportunity to review this very interesting article. The topic is highly significant --the article addresses the compelling issue of denial within the rapidly expanding field of study of climate obstruction.

Yet, the manuscript would benefit from further clarifications, particularly in the use of some key terms, while its engagement with parallel streams of literature will help strengthening the clarity and robustness of the main argument.

Title

Section 5.2 on Anti-environmentalism is crucial. Indeed, it could become part of the title itself as the theme of the far-right’s anti-environmentalism is much more central. Perhaps change the main title from ‘Defending the Western way of life’ (a rather underdeveloped argument within the article) into ‘The new far-right anti-environmentalism’ or, more bluntly, ‘Fighting against the environment’. I think this would much more concisely render the core of the article's argument and make improve its interdisciplinary accessibility.

Alternatively, if the authors choose to keep ‘Defending the Western way of life’ as the main title, then it may be worth going deeper into the colonial and imperial angles of notions such as Western or Westernisation, in which case an additional set of literature would be needed.

General comments

Is the term "conservative" correct when analyzing a political movement permeated by an extreme form of nationalism? Would another concept be more suitable to better convey the article's cogent message? More specifically, wouldn't far-right politics be a preferable term?

I note (and, I should add, find it hardly comprehensible) that the very notion of far-right is not mentioned once. Nor can I see any mention of the broader political right as such, except on line 743 when it appears in the context of the "Covid hoax" spread by right-wing (together with left-wing?) media. Here, the crucial political positioning of such pseudo-scientific claims on the left/right spectrum seems to be lost.

Throughout the text, the authors prefer to use the notion of "conservative movement”, yet a crucial question arises: is the term ‘conservative’ appropriate here? It is worth asking: “who conserves what?”. From Edmund Burke and Joseph de Maistre on, 'conservative’ refers to conserving the privileges of an elite and a few values associated with it, generally presented as ‘tradition’.

In other words, the term ‘conservative’ was the very term chosen by ‘conservatives’ themselves. Therefore, far-right politics(more specifically than right-wing politics, despite the strong association between the two) would be more appropriate, not least because of fascism’s historical association with denial, lies, and misinformation since its foundation by Benito Mussolini (see 'Eco-fascism: an oxymoron? Far-right nationalism, history, and the climate emergency', Frontiers in Human Dynamics, 6, 2024)

Indeed, the very notion that ‘Mitigation will arrest Western progress or development’ (65, 124, 589, 609, ) fully replicates both fascist ideology and far-right nationalist views (as above, see 'Far-right nationalism, history, and the climate emergency', 2024 ) — already most notably exemplified in the Belle Époque (late 1900s) by the interventionist and war-mongering futurist movement .

One common denominator of both contemporary far-right politics and historical fascism is the pervasiveness of nationalism, sometimes identified as ultra-nationalism, as works by Andreas Malm, following Roger Griffin (see White skin, black fuel: On the danger of fossil fascism. London: Verso, 2021).

I note that nationalism is never mentioned in the article, and, I think, this is the key missing link needed to assemble the pieces of the puzzle together. Indeed, until very recently, nationalism scholars themselves have been nearly oblivious to climate change, a meaningful absence that needs to be addressed. The first scholarly article to address the deep linkage between the two has only appeared in 2020: 'The ultimate challenge: Nationalism and climate change', Nationalities Papers, 2020

Comments and a few critical remarks

The correct observation that neoliberal and far-right's claim that "environmentalism is akin to totalitarian fascism" (642) is worth expanding. Yet, similar claims are sometime shared by circles and currents within the far-left, even among a few scholars, particularly political geographers (once more, see the first article mentioned above for a historical perspective).

As for reflexivity and anti-reflexivity (136-ff. 286-ff), this is fine-tuned with Dunlap’s 2014 Environmental Research Letters brief Perspective piece and finely expands on it. But how can it help understanding the complexity of the current political landscape? In this respect, and connecting again with the central nationalist component, the concept of “reflexive green nationalism" may perhaps be usefully incorporated. The reflexivity of "green nationalism" has been considered as a wholly alternative option vis a vis far-right’s anti-reflexivity.

The appropriate notion of pseudo-scientist appears more than once, but then why using the term “dissident scientists” (760)? The idea of free dissident is a badge of honour conferred upon those who spread misinformation, rather than rationally dissenting from science.

The term ‘misinformation’ only appears three times (191, 747, 754), far less than one should expect in an article devoted to analyse a movement based on the spread of misinformation. The term ‘disinformation’ also appears three times (741, 745, 750), but this is clearly dissectible form ‘misinformation’ (see ‘From climate change denial to war-mongering nationalism’, Cultures of Sustainability/ Culture della Sostenibilità, 31: 99-122.2023)

I find the far-right’s new "bête noire" of environmentalism as “the new socialist enemy to freedom’ (629) most relevant, central and inspiring. But it is well worth expanding on various dimensions of far-right politics, particularly utra-nationalism and neoliberalism — ’Capitalism and nationalism’, Ethnic and Racial Studies, 46 (8), 2023: 1685-1690. While not all nationalists are anti-environmentalists, and it may thus be possible to speak of "green nationalism" (see the recent booming literature on this), it is worth considering in depth the various linkages existing between far-right politics and anti-environmentalism.

The very notion of ‘movement’ applied to the CCCM also warrants closer scrutiny: depending on the emphasis and definition, the term ‘social movement’ may imply a spontaneous grassroots component. But this is an effort that may be worth pursuing in another article.

Minor comments

Line 30: “skeptical scientists that has successfully thwarted …”. The term “skeptical” has been criticized as it seems to grant an aura of respectability and even a scientific pedigree on those who disseminate antiscientific information. Yet, on lines 34 -37 you recognise their effort in “rejecting the basic findings of climate science” and that they “cast the integrity of climate science and scientists in doubt”. The term “anti-science” would be clearer.

35-36 The distinction between (trend denial), (attribution denial), (impact denial), and (policy denial) is very useful and essential.

51 -52 on the 97% consensus among climate scientists about human-caused greenhouse gas (GHGs) emissions, the percentage is apparently 100% - see ‘Scientists Reach 100% Consensus on Anthropogenic Global Warming, Bulletin of Science, Technology & Society, 2017, 37 (4): 183-184’.

60 when you rightly say that the CCCM was launched by fossil fuel corporations some references would perhaps be useful, while indications of the composition of the US Conservative Movement would also help. For instance, how far was the Tea party central here? See . The Tea Party and the Remaking of Republican Conservatism, Perspectives on Politics, 9 (1) 2011: 25-43 and. The Koch Network and Republican Party Extremism, Perspectives on Politics, 14 (3): 681-699, 2016.

Less important is ’Denial, deflection, and distraction: Neutralizing charges of racism by the Tea Party movement’, Mobilization, more superficial, but here the central notion of ‘distraction’ is briefly presented. In ongoing research, I have noted how ‘distraction’ can provide a broader and perhaps more explicative concept that climate obstruction itself.

67 Yes, it may be that “the roots of AGW denial thus go much deeper than protecting the fossil fuel industry”, but when did the movement begin? Wasn’t the fossil fuels industry that launched it? Why not using the notion of “fossil fuel complex”, which would include many other industrial actors.

Final recommendations

The underlying question is whether another set of data, however impeccable, may add substantially new information to the field. The field itself still lacks a broader historically grounded theoretical perspective about denial, disinformation and misinformation.

If the concepts used are inadequate or poorly explained, then the entire datasets associated with them risk loosing their scientific value, particularly if associated with interdisciplinarity, transdisciplinarity and cross-disciplinarity. That's why a conceptual deepening and a face-to-face assessment of the existing terminology should be a sine qua non requirement for publication. As the article is sufficiently robust and informative, this could be done quite easily without major revisions.

In short, this article is highly worth publishing and will doubtlessly have a high impact across disciplines. Such impact can be increased with some conceptual clarifications and amendments, particularly in two areas: first, the addition of two/three paragraphs highlighting the importance of nationalism and, secondly, attentively considering how and why the term ‘far-right’ is more appropriate than ‘conservative movement’ — at least in the sense in which both terms are intended beyond the USA.

Reviewer #3: I am a physical climate scientist rather than a social scientist, but I am extremely familiar with the climate denial environment. Overall, I don't see any problems with this paper. All of the results seem sound, and I have to say that what they accomplished required an enormous amount of work.

My only concern with the paper is that the books they analyzed have publication dates no later than 2010. Based on my own anecdotal experience, explicit climate denial—such as trend denial or attribution denial—has largely faded away since then. Now, prominent deniers such as Alex Epstein basically make arguments about what's best for humanity or Steve Koonin, who make value-based arguments ("The science is too unsettled for us to act") that sound like scientific arguments but are not, or Bjorn Lomborg, who makes arguments about the economics of climate change. None of these are really scientific arguments, although these people do their best to make them sound scientific.

My guess is that if you had a sufficiently long data set ending closer to today and conducted a time series analysis, you would see an interesting evolution of denial.

I don't have any specific suggestions for the authors, as I don't think it would be reasonable for me to demand they extend their analysis to include books published later. However, it would IMHO be useful to pursue that in future work.

**********

6. PLOS authors have the option to publish the peer review history of their article (what does this mean? ). If published, this will include your full peer review and any attached files.

If you choose “no”, your identity will remain anonymous but your review may still be made public.

**Do you want your identity to be public for this peer review?** For information about this choice, including consent withdrawal, please see our Privacy Policy .

Reviewer #1: No

Reviewer #2: No

Reviewer #3: No

**********

[NOTE: If reviewer comments were submitted as an attachment file, they will be attached to this email and accessible via the submission site. Please log into your account, locate the manuscript record, and check for the action link "View Attachments". If this link does not appear, there are no attachment files.]

While revising your submission, please upload your figure files to the Preflight Analysis and Conversion Engine (PACE) digital diagnostic tool, https://pacev2.apexcovantage.com/ . PACE helps ensure that figures meet PLOS requirements. To use PACE, you must first register as a user. Registration is free. Then, login and navigate to the UPLOAD tab, where you will find detailed instructions on how to use the tool. If you encounter any issues or have any questions when using PACE, please email PLOS at figures@plos.org . Please note that Supporting Information files do not need this step.

# Author comment: Author response to Decision Letter 1

**DOI:** 10.1371/journal.pone.0334544.r002

22 Aug 2025

We thank the editor and reviewers. Our responses to the reviewers are in bold.

Reviewer #1: The article describes an ambitious attempt to conduct a systematic qualitative analysis of a large number of books (over a hundred). This is, in itself, a convincing effort to uncover underlying themes in climate denialist literature. There arise a number of issues that need clarification. My overall assessment is between minor and major revision, and I would leave it to the editor to decide which category is more appropriate.

We appreciate the reviewers comments and suggestions.

One general comment is that, while the coding process done in stage one seems straightforward, it is unclear what the process exactly is in the second stage and how new themes emerged in this stage.

We have attempted to clarify this several ways in the ms. Part of the answer is found in the coding frame “what is the denial claim” and “what is the rationale” which are stages one and two respectively. Stage two also follows co-occurrence logic—within a chapter section (we realize this detail was missing and have added it now) what co-occurs with the denial claim. So, if an author denies that polar bears are threatened by climate change because s/he 1) says the number of bears is at a historic high and 2) polar bear scientists selling scare stories are corrupt—we take #1 to be superficial and #2 to be a more fundamental rationale because if scientists are thought to be corrupt none of their answers, say about the health of bear populations, can be trusted. Normally these two messages co-occur with each other in a chapter section because that section is targeting more specific things, like polar bear futures.

i. Corpus of Texts

What is the significance of the type of texts being analyzed (books)? I can intuit this, but perhaps this is something that needs explicit mention. Were books selected because, being treatise length texts, they are likely to contain whole narratives, completely written out, such that themes are fully developed? Are books deeper repositories of meaning, so that the "DNA" of climate denialist discourse are there to discern (but not in shorter texts such as blogs or articles)?

Our corpus was selected by 1) rejecting one or more of the three denial claims (trend, attribution, impact) and having an ISBN. All of our corpus is also in English. This means there is a wide degree of variance in the importance of each book. Some are from elite think tank policy insiders, some are self-published. We have added a statement explaining this. We did choose books as a target for the corpus because they are longer discourses and they confer a sense of authority to the author.

ii. First Stage

The two stage approach involves a deductive and inductive process. Isn't the deductive approach basically a thematic analysis, the a priori concepts being themes identified in previous literature? If so, perhaps the authors can clarify this.

Yes, it is a thematic analysis. We have added this language.

The findings from the first stage reinforce findings of previous investigations, namely four types of denial (trend, attribution, impact, policy). The discussion should answer the question: what was learned that was new or an addition to what previous works had identified? Or is the significance that these themes were almost universally found in systematic climate denial treatises (the books)?

Indeed, we confirm layers of findings in existing literature, so stage one does not add a whole lot to the literature, but is necessary in order to identify themes in stage two (i.e., what is discussed alongside the denial claim?). But we do take from this stage that the themes were almost universally found in the books.

Perhaps some discussion should go into sub-themes used in the thematic analysis, as these may provide a finer-grained discussion of the nature of each of the types of denial.

Thank you, we have added language to indicate that the quotes come from sub-themes. E.g., Section 4.2 we write: “Here are representative claims made by authors in the dataset, with each bullet point indicatinga sub-theme of the larger denial category”

The claim is made, later in the discussion of the first stage coding, that "We infer from this data that authors dismiss the role of GHGs in climate change in order to justify policy denial, because if the cause of warming is wrong, de-carbonization is unnecessary." But this higher-level, relational reasoning is something that emerges from stage two, isn't it (or perhaps I am not reading it correctly)?

We agree this is a premature statement at stage one and have removed it.

iii. Second Stage

On pages 15 and 16, there is a discussion of how coded text in stage one are subject to a relational type of coding in stage two. This section needs better explanation, as it is unclear how the process works. The discussion is confusing. It seems to suggest (but not at all clearly) that contiguity (of text) can be taken as logical necessity (i.e., that A � B). So, text (A) that constitutes some form of denial is succeeded by text (B) about climate science being a scientific hoax. So, what exactly is the connection drawn between A and B? Unclear what is being described… is the rejected science being equated to hypocrisy on the part of the scientists? What is the narrative, exactly? And is this logical connection between A and B what the authors later refer to as the theme of anti-reflexivity found in the second stage? This part of the discussion is confusing.

We have added the detail that the denial claim co-occurs within a chapter section and are thereby related and add meaning based on “the company they keep” (Firth 1968 citation added). We have also selected a different set of quotes from the same book (and have had to remove all the figures for copyright reasons) in figure 2 that hopefully lend better clarity to the process. We have also rewritten this section a bit to add clarity.

In addition, the two texts coded are contiguous within chapter sections, but are also connected semantically—authors tie the two sections together with their own language. We have also tried to clarify this more. For the anti-reflexivity finding, this comes in part from the move to stage two, but really from the combination of themes discovered in stage two. We revised to the following:

“Our nodes are connected by “proximities within a textual unit,” which are chapter sections in this case. We draw the connection between nodes through comments that directly and semantically tie one node to another. One node is always a statement of climate denial.” (p. 5).

A better description is needed of how exactly the themes (or narratives) of the politicization of science and anti-environmentalism found in the second-stage coding. Are these results of the relational coding described in pages 15 and 16? It is unclear, to this reviewer, how the inductive process works. Is it akin to coding in grounded theory? It is unclear how these two underlying themes emerged.

Take the paragraph below:

"Another example shows the anti-environmental theme in Fig. 3, which shows the denial (impact) claim, the connector in “But now we come to an additional point of overriding importance” and the issue of overriding importance is the “ulterior motives” of “the environmentalists, who are against anything that has to do with industrialization and comfort provided by technological progress” (Weber 1991, emphasis added)."

It is not clear what the analysis is, exactly. What exactly is the logical connection made between A and B --can this be stated definitively? Is A taken as proof of B? Or is the claim that B is an underlying theme to A? Does most of the second stage analysis involve such connection of two texts, and is the connector taken to be almost like a set operation, so that theme A is subsumed by the deeper theme, B?

We thank the reviewer for this overall observation because it indicates we have left some language out that should help. That language is that the authors semantically tie the two logics together themselves in their narrative within a chapter section—the denial claim and the meta-themes of anti-science and anti-environmentalism. We hope we have made this logic more transparent.

Perhaps a more thorough/explicit explanation is needed of how, for example, the anti-environmental theme was uncovered in the second stage of the analysis. Was this one theme among the others previously found (the four types of denial), or was it a meta-theme/meta-narrative that underlies the denialist text? How did the text in stage one lead to the identification of the anti-environmentalism discourse?

Anti-environmental is a meta-theme separate from the four CC denial claims/themes, and it was identified through recurring critiques of environmentalism in different ways (it’s a religion, it’s anti-human, anti-capitalist, etc….). The very same process identified anti-science as the other meta-theme. The consistency across the books and time was remarkable.

The identification of anti-reflexivity (amounting to anti-environmentalism and mistrust of the scientists behind impact science) is a significant finding, but was this not already identified in previous works (such as McCright and Dunlap, 2010)? What emerged from this analysis that adds to the previous account of anti-reflexivity?

Indeed, the reviewer is right that anti-reflexivity was identified by McCright and Dunlap in 2010. In that paper, the authors primarily analyzed anti-reflexivity in terms of non-decision making techniques and politics of the George W. Bush administration. Here, we add to this literature by covering a much longer time frame [1988? to 2010] and wider geographic representation with in-depth analysis of a large amount of data derived from a very different source—a large number of denial books--that we hope substantially adds a depth to the analysis that was not there before.

I feel that parts of the manuscript need to be rewritten for clarity. If the textual analytic method is novel, then clarity is needed even more so.

Thank you-- We have gone through and done an edit of the entire manuscript, looking for areas to improve clarity—including those you have raised.

Finally, there is a missing conclusion that is maybe alluded to but not stated definitively, that what is on the surface denial of a (scientific and social) movement is, at its root, a rejection of the alien other (whether labelled anti-capitalist, socialist, etc.). Which means that the different types of denial are contrived, masking a deeper agenda.

We have re-written the conclusion, hopefully answering this point. We agreed there needed to be a better summary. Here is part of it:

Anti-reflexivity is a powerful defensive mechanism employed by dominant interests to protect power and privilege ensconced in global capitalism fueled by cheap hydrocarbons and is consistent with far right nationalism, the protection of “petro-masculinity,” and white power. Dagget writes: “For many, extracting and burning fuel was a practice of white masculinity, and of American sovereignty, such that the explosive power of combustion could be crudely equated with virility” [15]. Likewise, Anshelm and Hultman [89] report, “These climate sceptics tried to save an industrial society of which they were a part by defending its values against ecomodern hegemony” (p. 84). Allowing meaningful restrictions to fossil fuels endangers the power of petro-privilege.

Hultman et. al. agree: “…far-right nationalism and AGW denialism [are] based upon the ideological similarities in viewing the world from an industrial masculinities viewpoint, not wanting to let go of the colonial extractive logic that has served these men well, but violated the planet” [90]. As Malm and the Zetkin Collective [14] show, following Griffin [91], far-right ideology, including fascism, has a historical relation with denial, lies, and misinformation since Mussolini. This helps us theoretically ground misinformation (not intended to deceive) and disinformation (intended to deceive) as forces that are capable of redistributing power and obscuring alternatives, “as when a traveler is falsely told a bridge collapsed” [92] notes Bok, and in this case our bridge is limiting fossil fuels to preserve a habitable planet.

We thank the reviewer for their considerate effort to help us improve this paper.

Reviewer #2: Thank you for the opportunity to review this very interesting article. The topic is highly significant --the article addresses the compelling issue of denial within the rapidly expanding field of study of climate obstruction.

Yet, the manuscript would benefit from further clarifications, particularly in the use of some key terms, while its engagement with parallel streams of literature will help strengthening the clarity and robustness of the main argument.

Title

Section 5.2 on Anti-environmentalism is crucial. Indeed, it could become part of the title itself as the theme of the far-right’s anti-environmentalism is much more central. Perhaps change the main title from ‘Defending the Western way of life’ (a rather underdeveloped argument within the article) into ‘The new far-right anti-environmentalism’ or, more bluntly, ‘Fighting against the environment’. I think this would much more concisely render the core of the article's argument and make improve its interdisciplinary accessibility.

Alternatively, if the authors choose to keep ‘Defending the Western way of life’ as the main title, then it may be worth going deeper into the colonial and imperial angles of notions such as Western or Westernisation, in which case an additional set of literature would be needed.

We thank the reviewer here for observing that the title needs some repurposing. We are now using the title: “Foundations of Climate Change Denial: Anti-Environmentalism and Anti-Science”

General comments

Is the term "conservative" correct when analyzing a political movement permeated by an extreme form of nationalism? Would another concept be more suitable to better convey the article's cogent message? More specifically, wouldn't far-right politics be a preferable term? This ignores the changes over decades in the CCCM. I don’t think that ‘far-right’ forces led the CCCM in the first two decades. The CoC and trade associations, e.g., were not part of a ‘far-right’ movement. But the CCCM was opposed by F

We appreciate this overall point, nationalism is clearly in our data but we did not frame it that way. We have gone through the paper and worked to see where “far-right” and nationalism can properly be added.

Here is part of our treatment:

The CCCM was launched by fossil fuel corporations and other corporate actors dependent on fossil fuel use to protect their interests and was quickly joined by the US Conservative Movement well established in the literature (Beder, 1999; A. McCright & Dunlap, 2000; Union for Concerned Scientists, 2015). This effort has evolved over time from mainstream conservative efforts (e.g., protecting markets from regulation) to now include more far-right programs. In the US this started with the Tea Party movement (Skocpol, 2014). The literature indicates that the climate denial connection to the far-right include white power (whether stated or implied) (Blee & Yates, 2015; Lichtman, 2008), authoritarian tendencies (Bryant & Farrell, 2024) as well as specific far-right alliances to the fossil fuel industry (Krange, Kaltenborn, & Hultman, 2019) of contemporary climate denial (Daggett, 2018; Malm & Collective, 2021).

Ekberg writes that climate denial has “clear signs of alliance with far-right actors”(Ekberg, Forchtner, Hultman, & Jylhä, 2022). Literature characterizing the “far-right” (Pirro, 2023), argues that the label “conservative” underappreciates the AGW denial is part of the CCCM’s reactionary effort to protect the status quo against progressive interests calling for change, especially in American society and protect the socioeconomic system based on a free-market economy with its promise of endless growth and unlimited progress-- to defend the “Western way of life” writ large (Jacques, 2006; Jacques, Dunlap, & Freeman, 2008; Klein, 2015). It is increasingly recognized that the roots of

Attachment Submitted filename: Reviewer comments.docx

# Peer review: Decision Letter 1

**DOI:** 10.1371/journal.pone.0334544.r003

**Authors:** Rahul Sambaraju

30 Sep 2025

Foundations of Climate Change Denial:  Anti-Environmentalism and Anti-Science

PONE-D-24-48750R1

Dear Dr. Jacques,

We’re pleased to inform you that your manuscript has been judged scientifically suitable for publication and will be formally accepted for publication once it meets all outstanding technical requirements.

Within one week, you’ll receive an e-mail detailing the required amendments. When these have been addressed, you’ll receive a formal acceptance letter and your manuscript will be scheduled for publication.

An invoice will be generated when your article is formally accepted. Please note, if your institution has a publishing partnership with PLOS and your article meets the relevant criteria, all or part of your publication costs will be covered. Please make sure your user information is up-to-date by logging into Editorial Manager at Editorial Manager®  and clicking the ‘Update My Information' link at the top of the page. For questions related to billing, please contact billing support .

If your institution or institutions have a press office, please notify them about your upcoming paper to help maximize its impact. If they’ll be preparing press materials, please inform our press team as soon as possible -- no later than 48 hours after receiving the formal acceptance. Your manuscript will remain under strict press embargo until 2 pm Eastern Time on the date of publication. For more information, please contact onepress@plos.org.

Kind regards,

Rahul Sambaraju

Academic Editor

PLOS ONE

Additional Editor Comments (optional):

Reviewer #1:

Reviewers' comments:

Reviewer's Responses to Questions

**Comments to the Author**

1. If the authors have adequately addressed your comments raised in a previous round of review and you feel that this manuscript is now acceptable for publication, you may indicate that here to bypass the “Comments to the Author” section, enter your conflict of interest statement in the “Confidential to Editor” section, and submit your "Accept" recommendation.

Reviewer #1: All comments have been addressed

**********

2. Is the manuscript technically sound, and do the data support the conclusions?

The manuscript must describe a technically sound piece of scientific research with data that supports the conclusions. Experiments must have been conducted rigorously, with appropriate controls, replication, and sample sizes. The conclusions must be drawn appropriately based on the data presented. 

Reviewer #1: Yes

**********

3. Has the statistical analysis been performed appropriately and rigorously? 

Reviewer #1: N/A

**********

4. Have the authors made all data underlying the findings in their manuscript fully available?

The PLOS Data policy requires authors to make all data underlying the findings described in their manuscript fully available without restriction, with rare exception (please refer to the Data Availability Statement in the manuscript PDF file). The data should be provided as part of the manuscript or its supporting information, or deposited to a public repository. For example, in addition to summary statistics, the data points behind means, medians and variance measures should be available. If there are restrictions on publicly sharing data—e.g. participant privacy or use of data from a third party—those must be specified.

Reviewer #1: Yes

**********

5. Is the manuscript presented in an intelligible fashion and written in standard English?

PLOS ONE does not copyedit accepted manuscripts, so the language in submitted articles must be clear, correct, and unambiguous. Any typographical or grammatical errors should be corrected at revision, so please note any specific errors here.

Reviewer #1: Yes

**********

6. Review Comments to the Author

Please use the space provided to explain your answers to the questions above. You may also include additional comments for the author, including concerns about dual publication, research ethics, or publication ethics. (Please upload your review as an attachment if it exceeds 20,000 characters)

Reviewer #1: I have read the revised ms, as well as the responses to the reviewers' comments. I feel that my own suggestions/comments have been satisfactorily addressed and have no further comments to add. I can recommend acceptance of the ms at this point.

**********

7. PLOS authors have the option to publish the peer review history of their article (what does this mean? ). If published, this will include your full peer review and any attached files.

If you choose “no”, your identity will remain anonymous but your review may still be made public.

**Do you want your identity to be public for this peer review?** For information about this choice, including consent withdrawal, please see our Privacy Policy .

Reviewer #1: **Yes: ** Raul P. Lejano

**********

# Editor report: Acceptance letter

**DOI:** 10.1371/journal.pone.0334544.r004

**Authors:** Rahul Sambaraju

PONE-D-24-48750R1

PLOS ONE

Dear Dr. Jacques,

I'm pleased to inform you that your manuscript has been deemed suitable for publication in PLOS ONE. Congratulations! Your manuscript is now being handed over to our production team.

At this stage, our production department will prepare your paper for publication. This includes ensuring the following:

* All references, tables, and figures are properly cited

* All relevant supporting information is included in the manuscript submission,

* There are no issues that prevent the paper from being properly typeset

You will receive further instructions from the production team, including instructions on how to review your proof when it is ready. Please keep in mind that we are working through a large volume of accepted articles, so please give us a few days to review your paper and let you know the next and final steps.

Lastly, if your institution or institutions have a press office, please let them know about your upcoming paper now to help maximize its impact. If they'll be preparing press materials, please inform our press team within the next 48 hours. Your manuscript will remain under strict press embargo until 2 pm Eastern Time on the date of publication. For more information, please contact onepress@plos.org.

You will receive an invoice from PLOS for your publication fee after your manuscript has reached the completed accept phase. If you receive an email requesting payment before acceptance or for any other service, this may be a phishing scheme. Learn how to identify phishing emails and protect your accounts at https://explore.plos.org/phishing.

If we can help with anything else, please email us at customercare@plos.org.

Thank you for submitting your work to PLOS ONE and supporting open access.

Kind regards,

PLOS ONE Editorial Office Staff

on behalf of

Dr. Rahul Sambaraju

Academic Editor

PLOS ONE
